# Viral Membrane Fusion and the Transmembrane Domain

**DOI:** 10.3390/v12070693

**Published:** 2020-06-27

**Authors:** Chelsea T. Barrett, Rebecca Ellis Dutch

**Affiliations:** Department of Molecular and Cellular Biochemistry, University of Kentucky, 741 South Limestone Street, Lexington, KY 40536, USA; chelsea.barrett@uky.edu

**Keywords:** viral fusion protein, transmembrane domain

## Abstract

Initiation of host cell infection by an enveloped virus requires a viral-to-host cell membrane fusion event. This event is mediated by at least one viral transmembrane glycoprotein, termed the fusion protein, which is a key therapeutic target. Viral fusion proteins have been studied for decades, and numerous critical insights into their function have been elucidated. However, the transmembrane region remains one of the most poorly understood facets of these proteins. In the past ten years, the field has made significant advances in understanding the role of the membrane-spanning region of viral fusion proteins. We summarize developments made in the past decade that have contributed to the understanding of the transmembrane region of viral fusion proteins, highlighting not only their critical role in the membrane fusion process, but further demonstrating their involvement in several aspects of the viral lifecycle.

## 1. Introduction

Enveloped viruses include many important pathogens, such as human immunodeficiency virus type 1 (HIV-1), Ebola virus (EBOV), influenza (IAV), measles (MeV), rabies virus, and severe acute respiratory syndrome coronavirus 2 (SARS-CoV-2). A critical early step for all enveloped viruses in the entry and infection process is the fusion of the viral membrane with a target cell membrane [1,2,3,4]. This process is mediated by at least one viral surface glycoprotein, often referred to as the fusion protein. Viral fusion proteins generally fall into one of three classes, based on structural similarities. However, despite these structural differences, the overall mechanism of how fusion proteins facilitate membrane merging is relatively conserved. Membrane fusion promoted by viral fusion proteins can occur either at the surface of the cell or within an endosome [5], and the location of this event is often determined by factors within the fusion protein or viral attachment protein ligand.

**Figure 1:**
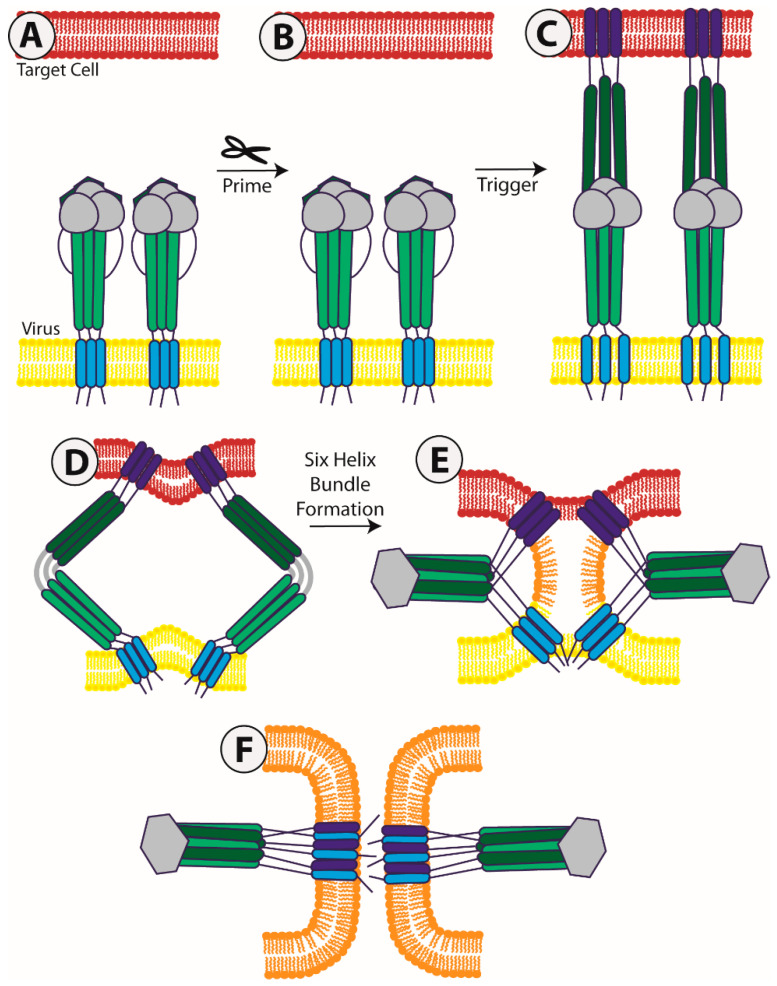
A model of viral membrane fusion function. This figure depicts a model of fusion mediated by a Class I viral fusion protein; however, related processes occur in the case of cClass II and III viral fusion proteins as well. (**A**) The fusion protein situates itself in the viral membrane (yellow). The first step of viral fusion is a priming event; in the case of Class I proteins, the protein itself undergoes the proteolytic processing to prime it for fusion. For Class II fusion proteins, it is a companion protein that gets proteolytically processed; (**B**) Once primed, the viral fusion protein remains in a metastable, pre-fusion conformation until it receives a triggering signal; (**C**) Upon receipt of the triggering signal, the protein extends out, forming a pre-hairpin structure, allowing for the fusion peptide or fusion loop (dark blue) to enter the target membrane (red); (**D**) This extended structure then begins to fold back on itself, bringing the N-terminal and C-terminal heptad repeats closer (dark green and light green, respectively), and in turn pulling the viral membrane and target membrane together; (**E**) As the N-terminal and C terminal heptad repeats zipper together to form a six-helix bundle, the target and viral membrane reach a hemi-fusion state, in which the outer leaflets have started to mix (orange); (**F**) Finally, the fusion peptide and transmembrane domain (light blue) come into close proximity to complete the merging of the two membranes and opening of the fusion pore. This final structure of a trimer of hairpins is a common conformation among all viral fusion proteins [9].

For most viral fusion proteins, two key steps are needed to allow for the large conformational changes which bring the viral membrane and cell membrane together [3]. The first is a priming step, a proteolytic cleavage by a cellular protease that exposes the highly hydrophobic fusion peptide (FP) or fusion loop (Figure 1A). This cleavage can occur in the *trans*-Golgi network as the viral protein traffics to the cell surface, in recycling endosomes after initial transport to the cell surface, upon receptor binding or viral particle endocytosis into a target cell, or as the virus is released from a cell. Additionally, this cleavage can occur to either the fusion protein itself (Class I fusion proteins) or an accessory viral protein (Class II). The second step is a triggering event. Triggering of the protein can be completed in a number of ways, including the fusion protein binding to a ligand, the viral attachment protein binding to a ligand and subsequent interaction of the attachment protein with the fusion protein, or exposure of the fusion protein to the low pH environment of an intracellular compartment (Figure 1B). Once a fusion protein has been activated and receives a triggering signal, the large, essentially irreversible conformational changes begin. The protein first extends away from the viral membrane to insert the FP or loop into the target membrane (Figure 1C). The merging of the viral envelope with the target cell membrane involves a high kinetic barrier, despite ultimately being a thermodynamically favorable reaction [2,3,6,7,8]. Because of this kinetic barrier, the energy contained in the fusion protein must help drive this process. While the depiction in Figure 1 is a Class I fusion protein, all the classes of viral fusion proteins share a similar end to the fusion pathway. This similarity arises during the pre-hairpin intermediate, where all fusion proteins studied to date share a homo-trimeric association [9]. The subsequent steps of fusion are hypothesized to involve a refolding of the protein back on itself to bring the fusion peptide and transmembrane regions into close proximity, and thus the two opposing membranes together (Figure 1D–F). During the membrane merger, there is likely first a hemi-fusion state between the viral and target cell membrane (Figure 1E), which then continues to a complete integration of the membranes resulting in the formation of a fusion pore which allows the genetic material of the virus to enter the cell (Figure 1F). Structures for the pre-fusion and post-fusion forms of multiple viral fusion proteins have been published [10,11,12,13,14,15,16,17,18,19,20,21,22,23,24], and the intermediate steps of this process, while previously unknown, are now being analyzed in a number of single particle studies, some of which will be discussed later [25,26,27,28,29]. Due to this intricately orchestrated process, studying all regions of these viral fusion proteins is critical for understanding the overall mechanism and forces that drive these processes.

The transmembrane domain (TMD) of viral fusion proteins remains one of the more poorly understood components of the membrane fusion process. Over time, the views of fusion protein TMDs have evolved as the field has shifted from considering them as simple membrane anchors to active players in membrane fusion [30]. In the 1990s, several seminal studies on influenza hemagglutinin (HA) and parainfluenza virus fusion proteins demonstrated that replacing this region of the protein with a glycosylphosphatidylinositol (GPI) membrane anchor resulted in loss of fusion but not outer leaflet mixing [31,32,33,34], suggesting the need for a proteinaceous membrane-spanning region for the hemi-to-full fusion transition. Subsequent studies analyzed sequence-specific requirements by creating chimeric fusion proteins in which the native TMD was replaced and found that some fusion proteins have sequence requirements while others do not [35,36,37,38,39,40]. Additional results suggested that there is a TMD length requirement for viral fusion proteins, as the TMD of these proteins needs to be long enough to span both leaflets of the viral envelope to facilitate the fusion process [35]. These studies suggest that TMDs of these viral fusion proteins play several functional roles in viral entry, including a role in the hemi-fusion to full-fusion transition, in promotion of outer leaflet mixing, and in fusion pore enlargement. Recently, significant advances have been made in understanding TMD interactions and the role of the TMD in fusion protein stability, structure, and function. In this review, we will discuss findings from the last decade that broaden our knowledge of the viral fusion protein TMD and the important role it plays in the overall membrane fusion process.

## 2. Class I Fusion Proteins

Some of the most studied viral families, including orthomyxoviruses (IAV), paramyxoviruses, retroviruses (HIV), coronaviruses, filoviruses, and arenaviruses, possess Class I fusion proteins. Class I fusion proteins exhibit a homo-trimeric association in both the pre-fusion and post-fusion states, and most of the secondary structure of these proteins is α-helical in both states. While these fusion proteins have been extensively studied for decades, there have been numerous advances on their mechanisms of action in the past ten years including work aimed at structurally and functionally characterizing the TMDs.

### 2.1. Influenza

The fusion protein of IAV, also known as hemagglutinin (HA), is one of the best studied viral fusion proteins. HA is a homo-trimeric protein that requires proteolytic processing to cut the protein into two subunits, HA1, important for binding to target cell receptors, and HA2, which facilitates membrane fusion. Previous work on the TMD of HA has implicated this region as playing a functional role, as there is a specific amino acid length requirement for this region and it was shown to be critical in late stage aspects of membrane fusion, such as fusion pore formation and enlargement [31,32,35,41,42]. Recent studies have continued to elucidate the important role of the HA TMD in the function of the full-length protein. In this section, we will discuss the contemporary findings that provide insight into the structure of the HA TMD, its role in the dynamic intermediates, the post-fusion conformation, and in HA interactions with the membrane environment during the fusion process.

**Figure 2:**
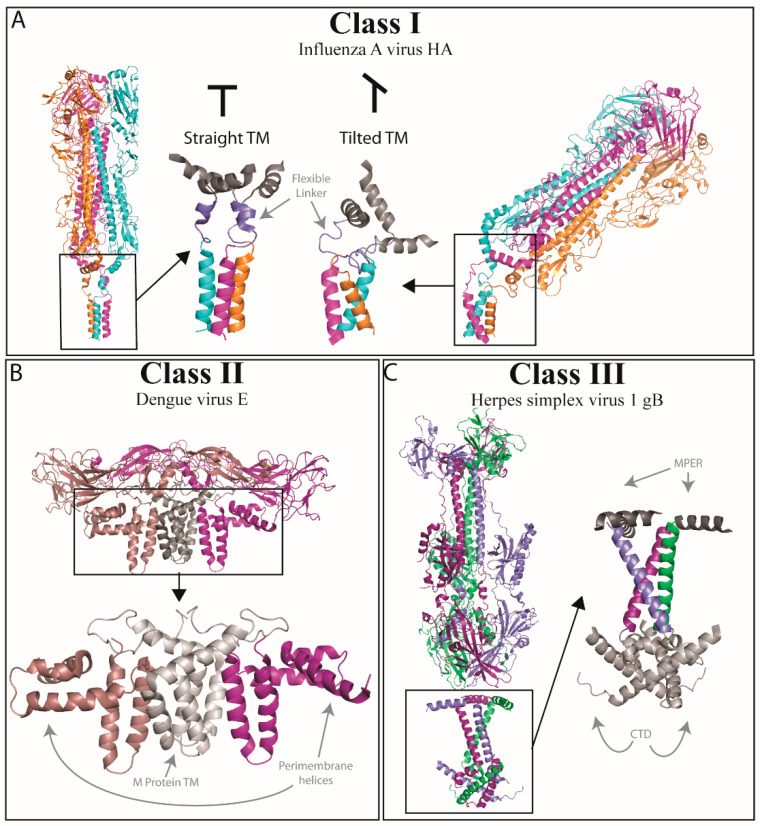
Full-length structures of viral fusion proteins. While the ectodomain structures of numerous viral fusion proteins have been solved, only a few solved structures of full-length viral fusion proteins, including the transmembrane domain (TMD) have been solved. (**A**) Full-length influenza hemagglutinin (HA) was solved in 2018 [51]. Two structures were published, 6HJQ (far left) and 6HQR (far right). The first is HA in its pre-fusion conformation, and the ectodomain is in line with the TMD helices (middle left, zoomed in). In the second structure, the ectodomain is tilted 52° with respect to the TMD helices (middle right, zoomed in); this likely represents a scenario that is an intermediate state of fusion. In each zoomed-in area, a linker region is indicated (slate), and this region remains flexible to help compensate for this tilt. (**B**) In 2013, the structure of full-length Dengue virus E protein was solved [53]. This structure showed the hetero-tetramer complex of two E proteins (light and dark purple) and 2 M proteins (gray) with their respective TMD helices (3J2P). Flavivirus E proteins have two TMDs that have extensive hydrophobic interactions between them. The structure also includes three peri-membrane helices that lie on the outer surface of the viral membrane, approximately perpendicular to the TMD helices. (**C**) Full-length Herpes simplex virus 1 gB protein was published in 2018 [54]. This structure (5V2S) shows the three TMD helices situated in a triangular teepee structure; the MPER (dark grey) is a helix that lies almost perpendicular to the orientation of the TMD helices. The solved structure also includes a large portion of the cytoplasmic tail (CTD, light gray). Each CTD has two helices, the first of which is a small helix that links to a larger helix which then angles back towards the inner leaflet of the viral membrane. Because of the orientation, these CTDs may act as a clamp that assists in holding the gB TMDs in specific conformation, and the angle of the CTD may work in concert with the TMD helices to dictate the overall conformation of the protein. Figure made with PyMOL (Schrödinger®, Neo York, NY, USA).

While structures of the ectodomain of HA have been available for several decades [11,12,43,44,45,46,47,48,49,50], the first structure of the full-length HA protein, including the TMD, was published in 2018 [51]. When compared to previously published structures of the HA ectodomain alone, the ectodomain of the full-length HA structure is very similar, indicating that inclusion of the TMD does not profoundly affect the ectodomain conformation. Interestingly, the TMD was found at angles between 0° and 52°, with respect to the ectodomain region (Figure 2A), revealing the presence of a flexible linker region between the ectodomain and the TMD. This flexible linker region consists of a conserved glycine followed by a small five-residue α-helix and a four-residue extended chain (residues 175–184). When the structure was solved in complex with a FISW84 Fab, this angle was restricted to 20° or less. Analysis of the structure showed that the base of the ectodomain lies in a horizontal orientation relative to the membrane. Conserved glycine residues at the C-terminus of the ectodomain and the end of the helix in the flexible linker allow for side chain turning to facilitate flexibility in this region. A conserved isoleucine begins the bundled α-helices of the TMD, which extend for 16 residues to a conserved leucine, then glycine residue. Within this α-helical bundle, a tyrosine residue provides a linkage point between the helices. Although the TMD extends seven residues past the conserved glycine, the helices become less ordered in this region. It is likely that the three conserved glycine residues are critical to allow the large degree of tilt of the TMD with respect to the ectodomain observed in the solved structures. Furthermore, in these different tilted forms, the helices in the TMD maintain their secondary structure but rotate with respect to the other helices in the trimeric bundle. In all the tilted forms, there are consistent contacts with the central tyrosine, indicating this may be crucial for maintaining inter-helix contacts.

This independent movement of the individual TMDs of HA is consistent with previous molecular dynamics simulations of the TMD in isolation which found that, when inserted into a DMPC lipid bilayer, there was no direct contact observed between multiple TMDs [52]. Additionally, a single HA TMD peptide exhibited a tilt angle of about 60° in the membrane in molecular dynamics simulations. When three TMD peptides were present, the tilt angle increased by 10°, and the peptides arranged in a triangular manner, similar to the arrangement in the full-length structure [51]. When mutations were introduced into the TMD peptide, the helicity of the peptides was altered, but no overall effect was seen on the tilt angle of the peptide in the membrane. These studies indicate that the tilt of the TMD of HA with respect to the membrane may play a role in membrane fusion, as different angles may be needed to compensate for the large conformational change experienced by the ectodomain of the protein. Additionally, the finding that the TMD peptides alone arranged into trimers suggests that the TMD may play a role in the overall trimerization of the protein.

In the cascade of HA viral membrane fusion, there are likely a series of intermediate protein arrangements between the metastable pre-fusion HA conformation and the post-fusion form, but these have been difficult to capture. Recent studies have succeeded in identifying protein intermediates in the fusion process [55], and two have used the full-length protein [26,56], providing important new information. The first used cryo-microscopy and cryo-tomography to visualize viral particles fusing with liposomes upon low pH treatment [56]. Images captured showed that prior to full viral-liposome fusion, the viral particle has several contacts with the liposome membrane. These contacts were seen as thin, continuous lines between the viral particle and the liposome, with the length of these lines consistent with an extended conformation of the HA protein, with the TMD still embedded in the viral particle and the FP in the liposome membrane. Around these zones, the liposome membrane exhibited a dimpling effect out towards the viral particle, potentially as a result of multiple FP insertions into the target membrane. Additionally, bent versions of the extended structure were observed, consistent with the protein folding back on itself as it moved towards the post-fusion structure. Radiating outward from the central dimpled region were dense bars of HA protein. These bars appeared even before full fusion pore formation, which may be the result of either already folded back HA proteins or HA proteins that triggered but did not insert in the target membrane. This work confirms the presence of a full extended intermediate of HA along the fusion cascade and demonstrates several other intermediate forms.

Another study analyzed conformational changes of the protein that occur prior to the full extension intermediate of HA. To analyze the HA protein in a single molecule study, a Forester resonance energy transfer (FRET) HA protomer, which includes the full-length HA from the strain H5N1, with its TMD, was created [26]. The addition of two fluorophores to the HA2 subunit allowed for reporting of a pre-fusion conformation (high FRET) or a post-fusion conformation (low FRET). Analysis of this tagged HA protein within the context of a single viral particle found that even at neutral pH (pH = 7.0), the protein spent time in three distinct conformations, a high FRET, an intermediate FRET, and a low FRET state. As the pH was decreased from neutral pH, the HA protein demonstrated an increase in occupancy of the low FRET state in a stepwise manner. The amount of protein found in the intermediate state stayed consistent regardless of the pH. Interestingly, samples that were exposed to low pH for short periods of time were able to revert back to high FRET states upon return to neutral pH. However, those that were exposed to low pH for extended times (30 minutes or more), were unable to return to high FRET states. This indicates that the protein may sample low pH conformations prior to irreversibly converting to the post-fusion conformation. FRET experiments were also completed in the presence of stalk-targeting antibodies, the HA receptor sialic acid, and a target membrane. Co-expression with stalk-targeting antibodies prevented transition of the protein to the low FRET state while increasing the occupancy of the protein in both the high and intermediate FRET states. The presence of sialic acid increased the overall kinetics of the conversion between high and intermediate to low FRET states, while the presence of a target membrane increased the amount of protein that was found in the irreversible low FRET state. This suggests that there is a breathing movement of the full-length HA protein prior to the extended intermediate in the fusion cascade. This dynamic movement may help temporally control the fusion process by allowing HA to sample its environment, thus ensuring conditions are correct for a full fusion event to occur. Movement of the TMD with respect to the ectodomain, conferred by the flexible linker region [51], may be important for these dynamic intermediates to occur.

In the post-fusion form of the HA protein, the FP and the TMD are in close proximity. Previous work has demonstrated that these regions can form a complex within the membrane environment [57], though the role of this complex is unknown. To address this, a recent study examined the effect of the HA FP and TMD both alone and together on membranes using electron spin resonance [28]. Both the FP and TMD alone have an ordering effect on several different types of membranes, with a synergistic effect observed when both the TMD and FP are present in the same membrane. When FP is alone, pH affects the membrane ordering, but the FP-TMD membrane ordering is not affected by changes in pH. While it has been previously shown that the TMD alone induces distinct micro-domains in the membrane [58], the FP-TMD complex is also able to induce these, to a greater extent than the TMD alone [28]. To further examine the FP-TMD relationship, mutations known to affect membrane fusion were made to FP residues. When an FP with a G1S mutation, known to block fusion at the hemi-fusion step [59], is present, some lipid ordering still occurs, but no synergistic effect was observed when the wildtype TMD peptide was added. In contrast, addition of the fusion-blocking mutation G1V to the FP resulted in complete loss of lipid ordering, suggesting this glycine residue in the FP is critical for the FP-TMD complex formation. A mutation at Y14, also previously shown to block fusion, was still able to induce membrane ordering when the TMD was present, suggesting that this mutation does not block the FP-TMD interaction. In the TMD, mutations K183E and L187A have been demonstrated to abolish the membrane ordering effect of the TMD itself [58]. Analysis of these mutations using electron spin resonance to measure membrane ordering in the presence of the FP suggested that L187 played a key role in the FP-TMD interaction, while the mutant K183E did not. This suggests that the FP-TMD interaction is strongly influenced by the N-terminal portion of the FP and the hydrophobic segment of the TMD. Furthermore, the insertion depth into the membrane of the N-terminus of FP was found to increase in the presence of the TMD, again supporting an interaction between the two.

Contrary to the work described above, a study from 2018 on the FP-TMD did not provide evidence of complex formation [60]. Using hydrogen–deuterium exchange mass spectrometry (HDX-MS), Ranaweera et al. studied the full-length HA2 subunit or the HA2 ectodomain with either the FP or the TMD present. Extensive exchange was observed when the FP region was present in both the full-length and the truncated protein, while the TMD demonstrated very little, supporting a model in which the FP lies along the membrane face a portion of the time, allowing for exchange, while the TMD traverses the membrane. The results did suggest, however, that the orientation of the FP and TMD with respect to each other and the HA ectodomain may play a role in creating positive membrane curvature to help with fusion pore expansion. The contrasting results from these two studies warrant further research into the relationship between the HA FP and TMD.

Since the TMD does not exist in isolation but in the context of the membrane environment, several studies have examined the relationship between HA and the lipids of the membrane [61,62,63,64]. HA contains two raft targeting signals, one on the outer leaflet of the TMD and one at the interface of the TMD and cytoplasmic tail [65,66,67,68,69]. Mutation of the signal in the outer leaflet of the TMD caused slower transport through the Golgi, whereas mutation of the second signal did not delay transport [61], and both mutants displayed reduced association with rafts at the plasma membrane. To further delineate the relationship between membrane lipids and HA, a study analyzed the effect of mutating a conserved cholesterol binding motif, YKLW, found at the interface of the TMD and the flexible linker in HA proteins from the phylogenetic group 2 [62]. This work demonstrated cholesterol directly binds to HA through this region. Mutation of this motif to alanines resulted in a reduction in viral replication, HA and cholesterol incorporation into viral particles, and HA fusion activity. This mutation appears to specifically affect the extent and kinetics of lipid mixing during the hemi-fusion state, suggesting that an HA TMD-cholesterol interaction is critical for this aspect of membrane fusion. However, work completed in 2015 suggests these interactions may not be critical for all subtypes of HA. Using high-resolution secondary ion mass spectrometry on stable cell lines expressing HA (H2 subtype, phylogenetic group 1), the colocalization of HA with common membrane lipids was assessed [63]. HA demonstrated little colocalization with either cholesterol or sphingolipids, suggesting HA, at least from this subtype, does not associate with membrane raft domains. These contrasting data may be due to the difference in HA subtypes used, but further exploration of the interactions of HA with the surrounding membrane is warranted.

Recent work has illuminated the influenza HA TMD structure and has characterized a flexible linker region that lies between the ectodomain and the TMD [26,51,52,56]. Additionally, studies have shown that some subtypes of HA bind cholesterol in the TMD, suggesting that, together with the FP, the TMD plays a role in the membrane manipulation needed to facilitate the merging of the viral and target membrane [28,60,61,62,63]. While this section reflects the immense amount of work completed on HA TMD over the past several years, it is clear from the number of conflicting studies that more work needs to be completed. Though there is some conservation of the TMD of different HA subtypes [70], the TMD of each subtype may have its own unique properties that need to be investigated.

### 2.2. Human Immunodeficiency Virus (HIV)

The fusion protein of HIV is known as the Envelope protein (Env). Similar to the influenza HA protein, HIV Env (gp160) consists of two subunits, a gp120 receptor-binding domain and a gp41 membrane-spanning domain that mediates viral fusion. There is a high degree of conservation in the TMD of gp41 from different HIV strains, and that conservation was first used to implicate the TMD as more than just a membrane anchor [71]. Similar to influenza HA, there has been extensive work over the past decade on the gp41 TMD. In this section, we review studies which illuminate the structure of the TMD, the dynamic nature of FP-TMD interactions, the role of the TMD region as a modulator of immune function, and the role of the TMD in overall protein trafficking.

The number of structural studies of the TMD or TM proximal regions of Env gp41 exemplify the considerable amount of work recently completed in this area. In the past decade alone, there have been studies examining the TMD in isolation [72,73,74], the TMD with the membrane proximal external region (MPER) [75,76,77], the gp41 ectodomain with the FP proximal region and the MPER [78], as well as a full-length structure of gp41 (including the TMD) [79]. All-atoms molecular simulation models and nuclear magnetic resonance (NMR) have been used to probe the structure of the HIV Env TMD in isolation [72,73,74]. These studies suggested that the TMD forms a closely assembled trimer [72,73]. The conserved residue R696 serves as a midpoint between two distinct domains in the TMD, an N-terminal coil-coiled domain, and a C-terminal hydrophilic core domain. The N-terminal coil-coiled contains a GXXXG oligomerization motif, but the data showed that only the first G in the motif lies at the interface of the trimer, while the other lies away from it [72]. This suggests that for trimer formation using a GXXXG motif, only the first G is essential. Further analysis demonstrated that the N-terminal half of the TMD appeared to be less structurally stable than the C-terminal half [73]. These studies did not report on the orientation of the TMD with respect to membrane, though an all-atoms molecular dynamics simulation determined that a single protomer of the TMD formed a stable tilted α-helical region [74].

To situate the TMD in relation to the ectodomain of gp41, a series of structural studies were completed with peptides containing both the TMD and the MPER of gp41 [75,76,77], but interestingly the findings vary. The earliest study found that the MPER and N-terminal portion of the TMD create an aligned α-helix, while the C-terminal region of the TMD is also α-helical, but is not in frame with the rest of the protein [77]. Subsequent work demonstrated that the MPER exists in two distinct α-helices which are connected to the TMD through a kink at residue K683 [75]. In agreement with this, another study demonstrated a turn at residue 683, but their data suggested that both the MPER and TMD consisted of a single α-helical region each [76]. These discrepancies may be due to the use of bicelles in the first two studies [75,77] and phospholipid bilayers in the latter [76], differences in the peptide purification method, and the use of a tag on the peptide [77], or the differences may reflect different states of these regions along the fusion cascade. Regardless, further work is needed to delineate the structure of these regions in context to each other, and studies using the full-length protein may help better understand the relationship of these regions.

Work that includes the entire full-length protein, or just portions of the ectodomain, does not yet resolve these questions [78,79,80,81]. When the entire protein was present, the TMD and MPER were unable to be resolved, suggesting either different conditions are needed for structural analysis or there is an increase in flexibility in this region when the entire ectodomain is present [79]. When all protein domains, other than the FP and the TMD, are structurally determined in the post-fusion form gp41, the ends of the FP proximal region and the MPER splay outward from each other [78], suggesting the FP and TMD may not be in close proximity to each other in the post-fusion structure. The structure of an MPER trimer in isolation also supports this by demonstrating a splaying out of the helices of the trimer as they approach the membrane [80]. Both of these studies also found some MPER insertion into the detergent micelle, suggesting that the MPER has some degree of interaction with the membrane.

During the viral-membrane fusion process, the ectodomain of gp41 undergoes a large conformational change, moving from a pre-fusion state and refolding to a post-fusion conformation. This change brings the TMD and FP in close proximity, similar to HA, but there is some debate as to whether these hydrophobic regions physically interact. One study demonstrated that gp41 FP- and TMD-derived peptides directly associated with each other and together were able to induce lipid mixing in membranes [82]. Work with a synthetized protein that included the FP, a small region of the ectodomain at the C-terminus of FP, MPER, and TMD with a short flexible region connecting the FP proximal region and the MPER, revealed that the FP has mostly β-sheet structure and is partially inserted into the membrane, while the TMD region is α-helical and traverses the membrane [83]. In contrast to the previous work, this study showed no evidence for FP-TMD interactions. These data, however, do suggest that protein conformations associated with a hemi-fusion intermediate step exist between the pre-fusion and post-fusion conformations of the protein.

While the dynamic nature of the gp41 ectodomain is apparent by the differences in pre-fusion and post-fusion structures, the dynamic nature of the TMD is just beginning to be uncovered. Work that replaced the TMD of gp41 with a TMD of another viral fusion protein or another membrane-spanning protein found that fusion inhibition occurred, likely due to alterations in the ectodomain conformation of the protein, suggesting differences in interactions within the TMD play a critical role in the overall protein conformation [84]. Further illuminating the dynamics of the TMD, several studies have investigated conformational changes that occur in the TMD during the fusion process, with many of these focusing, at least in part, on a mid-TMD arginine residue (R696). R696 is highly conserved among different HIV subtypes and has been implicated as critical for membrane fusion [85]. Molecular dynamic simulations suggest the position of R696 with respect to the membrane leaflets likely plays a role in facilitating the fusion event. R696 can snorkel to interact with the inner leaflet of the membrane, allowing for water penetration into the membrane and membrane thinning needed for membrane fusion [86]. Additional simulations investigated the relationship between cholesterol and R696 [87]. It was determined that R696 allows for water penetration in a variety of membranes, but cholesterol-containing membranes help localize the overall membrane thinning associated with this water penetration to the mid-span arginine residue, likely by regulating the tilt angle of the TMD relative to the membrane. There is evidence that R696 also acts in concert with the C-terminal hydrophilic core of the TMD to allow for water penetration into the membrane [88]. This concerted action is consistent with R696 snorkeling to the inner membrane leaflet (towards the C-terminus), allowing for membrane perturbations consistent with those needed to facilitate viral entry. There are two additional conserved basic residues (K683 and R707) in the TMD of gp41 [86]. These residues likely interact with the head groups of the outer and inner membrane lipids, respectively. Observations from membrane dynamics simulations conclude that these head groups anchor the TMD to the edges of the membrane so that when R696 snorkels, the pull on both of these residues also contributes to the membrane thinning.

HIV-1 infection of cells disrupts normal immune responses, allowing the virus to avoid detection. Both Toll-like receptor (TLR) activity and T cell receptor (TCR)/cluster of differentiation 3 (CD3) complex formation are down-regulated in HIV infection, and gp41 can disrupt TCR and CD3 complex formation to inhibit immune activity [89,90]. However, this disruption was only recently shown to be due to direct binding of the gp41 TMD with the TMD of both TCRs and CD3. These interactions occur within the membrane environment and specifically use the GXXXG motif found in the TMD of these proteins [91,92]. Down-regulation of TLRs was also found to involve interaction with the TMD of gp41 through the GXXXG motif, suggesting that this motif could play a role in other interactions that disrupt the immune response during an infection [93]. The isolated peptides from the gp41 FP region also interact with the TMD of TCRs through a similar motif, AXXXG, which suppresses TCR immune activity [90,94,95,96]. When the AXXXG motif was present in FP-mimicking peptides, lipid mixing could be induced, but when this motif was mutated, lipid mixing did not occur, suggesting this motif may be important in the transition from hemi-fusion to fusion pore formation during membrane fusion.

Induction of broadly neutralizing antibodies (bNAbs) is a critical part of current HIV vaccine strategies. bNAbs to the HIV Env protein have several different targets, one of which is the MPER region bordering the TMD. However, stabilized soluble trimer mimics of Env, termed SOSIPs, lacking a majority of the MPER and all of the TMD are commonly used in HIV vaccine development. Recent research has demonstrated that antibody binding differences may be dependent on which regions of the Env protein are present [97,98]. A direct comparison of a SOSIP trimer to a full-length Env trimer showed that SOSIP trimers had less complex and less processed glycans compared to the full-length protein. Glycans are part of several binding epitopes for bNAbs, and the differences in complexity in SOSIP trimers resulted in lower binding affinity of these antibodies compared to the full-length Env. This comparison also revealed that the full-length protein had more conformational flexibility than SOSIPs and therefore exposed epitopes that also bound the non-neutralizing antibodies tested. Inclusion of the TMD with an MPER peptide has been shown to increase the binding affinity of bNAbs to these peptides, although there is conflicting data on whether a trimeric TMD further increases this affinity. One set of binding assays completed in nanodiscs suggests that the addition of a trimeric-TMD recapitulates the bNAb binding of native-like Env protein [97], while another suggests inclusion of a single MPER-TMD peptide in each nanodisc increased the percentage of antibody bound to that peptide [99]. It has been shown by both NMR modeling [77] and crystallography with molecular dynamics simulations [100] that bNAbs targeting the MPER region of gp41 bind residues within the TMD as part of their epitope, explaining why the presence of the TMD increases binding affinity. Additionally, analysis of one specific MPER bNAb demonstrated that this antibody also interacted with membrane lipids [100]. This suggests that the conflicting results on the effect of TMD oligomerization on MPER-TMD bNAb binding may be due to the presence or absence of certain lipids. Taken together, these studies suggest that efficient testing and analysis of bNAbs targeting Env MPER should include the TMD and potentially a representative membrane environment. Beyond just enhancing the testing efficacy of MPER-targeting bNAbs, utilizing versions of the full-length protein may be a way to further improve current vaccine candidates.

During an HIV infection, Env is synthesized in the endoplasmic reticulum and traffics through the Golgi and secretory pathway to reach the plasma membrane [101]. The TMD of the Env (gp41) protein has been implicated in this protein trafficking. As previously described, the TMD contains both a GXXXG oligomerization motif and a mid-span arginine that are highly conserved. When the distance between the last G in the GXXXG motif and the mid-span arginine is increased by the addition of an alanine residue, a defect in membrane fusion is seen [102]. This was shown to be due to a defect in protein transport through the endoplasmic reticulum (ER) and Golgi. This transport delay may be due to disrupted contacts of the individual trimer TMs with each other or through disruption of protein-membrane interactions. Further analysis of this region demonstrated that R696 does not confer a strict ER localization on the gp41 protein, despite the presence of charged residues within a TMD being a well-recognized ER localization motif [103]. Therefore, other elements in the HIV Env TMD, including its length, override the potential retention signal.

It is clear that the HIV gp41 TMD plays an essential part in the structural stability, function, and trafficking of the gp41 protein. This recent work has demonstrated that the gp41 TMD is critical for processes such as virus-to-cell fusion, immune modulation, antibody recognition, fusion protein trafficking, and several aspects of the membrane fusion cascade. These studies also continue to uncover vaccine and antiviral targets for this important human pathogen by understanding key molecular and cellular interactions.

### 2.3. Paramyxoviruses

Paramyxoviruses have Class I fusion (F) proteins that require both a proteolytic cleavage event and receptor binding to facilitate fusion. However, unlike the previously discussed IAV HA and HIV Env proteins, the receptor binding function of this process is executed by a separate viral surface glycoprotein, the attachment protein (HN, N, or G). Work in the last decade has demonstrated a role for the TMD in the overall structure of the fusion protein. In addition, it has also been shown to be important for pre-fusion stability, membrane fusion, post-fusion FP-TMD interactions, fusion protein trafficking, and viral particle assembly.

Crystal structures have been solved for the ectodomain portions of several paramyxovirus fusion proteins [10,16,17,22,23,24], but structural insights into the TMDs of these proteins remain limited [104,105]. Solid-state NMR analysis of isolated TMD peptides of parainfluenza virus 5 (PIV5), separate from the rest of the protein, found that portions of the TMD display some membrane-dependent conformational plasticity. Both ends of the TMD adopt a β-strand conformation in phosphatidyl ethanolamine (PE) rich (negative curvature) membranes but form a continuous α-helix with the central portion of the peptide in phosphatidyl choline (PC)/cholesterol rich membranes [104,105]. These flexible regions of the TMD are rich in β-branched residues. This indicates both termini of the TMD could play a role in the membrane perturbation needed to mediate membrane fusion. The central portion of the TMD, however, was shown to form a core α-helical region that associates as a trimer with neighboring TMDs regardless of the membrane composition. This 12-residue, leucine-rich stretch may serve as the central trimerization domain needed for overall protein oligomerization.

Despite limited structural data, biochemical and biophysical studies have also probed the trimeric nature of paramyxovirus TMDs. Initial work that substituted the residues in the predicted TMD of PIV5 F with cysteine residues to induce disulfide bonds within the membrane demonstrated that the TMD of F existed as α-helices and formed a helical bundle with the other TMDs of the protein trimer [106]. Further analysis of the TMD helical bundle was completed using sedimentation equilibrium analytical ultracentrifugation (SE-AUC). Using this technique, the fusion protein TMD of Hendra, human metapneumovirus (HMPV) (now in the *Pneumoviridae* family [105,107]), and PIV5 were demonstrated to exist in a monomer–trimer or monomer–trimer–hexamer equilibrium when studied in isolation [108].

To examine the effect of this TMD association on overall protein folding and function, two common oligomerization motifs, a AXXXG motif [108] and a Leucine-Isoleucine Zipper (L-I Zipper) [109], were mutated in the Hendra F protein. Mutations of the glycine in the AXXXG motif led to a decrease in cell surface expression and a decrease in fusion activity at levels consistent with the reduced protein expression. Single alanine mutations of each residue in the L-I Zipper had varying effects on protein expression and fusion activity, suggesting that each has a unique role. However, when all four residues in the L-I Zipper were mutated to alanine, a decrease in the expression of the protein was shown, and the fusion activity of the protein was abolished. Further analysis with SE-AUC showed a 1000-fold decrease in the association constant in the monomer–trimer equilibrium, indicating TMD-TMD associations were destabilized when the L-I Zipper was altered [109]. A heat-induced triggering assay demonstrated that mutations which altered TMD-TMD association also led to a decrease in stability of the pre-fusion form of the Hendra F protein, suggesting TMD-TMD associations are important for holding the F protein in the prefusion conformation prior to triggering. Replacement of the TMD of Newcastle disease virus (NDV) with either related or non-related viral protein TMDs demonstrated alterations in conformation-specific antibody binding [110], suggesting that, similar to Hendra, specific TMD-TMD associations are needed for the stability of the proper pre-fusion conformation of the fusion protein. Interestingly, mutation of the L-I zipper motif in the TMD of PIV5 to alanine had no effect on the total expression or pre-fusion stability of the protein and only a minor effect on surface expression, but fusion activity was abolished [106,111]. Taken together, these data suggest that the contribution of leucine zippers in fusion protein TMDs to the overall protein stability and function may be virus specific.

Following triggering, the F protein of paramyxoviruses undergoes large conformational changes that include insertion of the FP into the target membrane, followed by the protein refolding back on itself to facilitate formation of the six-helix bundle. While the details of the TMD throughout this process are still being investigated, there is clear evidence for an active role of the TMD and TMD-TMD interactions along the fusion cascade [105,106,110,111,112,113]. Illustrating the role of the TMD in the fusion process, replacement of the NDV F protein TMD with the TMD of a related viral fusion protein abolished fusion, including hemi-fusion intermediates, despite the chimeric fusion protein being expressed and cleaved at the cell surface [110]. This lack of fusion may be due to an inability of these proteins to form complexes with the NDV HN protein which is critical for membrane fusion, though other mechanisms are also possible.

To further probe specific residues of the TMD that are critical for fusion, several studies performed mutagenesis on paramyxovirus F protein TMDs and analyzed differences in fusion activity [106,108,109,111,112,113]. Alanine scanning mutagenesis found that β-branched or just branched amino acid residues at the C-terminus of the TMD appear to play an important role in fusion in both PIV5 [106] and Hendra [113]. Analysis of these branched residues in PIV5 demonstrated that mutating them likely blocks fusion during the hemi-fusion or fusion pore formation stages, indicating they may play a role in lipid mixing. This hypothesis is further supported by the structural analyses that revealed conformational flexibility found in the β-branched-rich TMD termini of PIV5 and showed this flexibility promotes changes within the membrane needed for fusion to occur [104,105]. Recent work also suggests that TMD-TMD associations in paramyxovirus F proteins are important for controlling the fusion cascade. Mutating the L-I Zipper motif in the F protein TMD completely suppressed fusion activity in both PIV5 and Hendra, despite there being cleaved protein at the cell surface in both cases [109,111]. Interestingly, introduction of disulfide bonds to prevent TMD-TMD dissociation also disrupts fusion in Hendra [112], as does introduction of disulfide bonds directly N-terminal to the TMD in PIV5 F [114]. These studies suggest that TMD-TMD association and dissociation must be intricately controlled for membrane fusion to occur.

The final steps of fusion involve a zippering together of the N- and C-terminal heptad repeat regions [2,3,115,116], and this refolding brings the FP and the TMD in close proximity to one another. Solid-state NMR analysis of the FP and TMD of PIV5 F in a lipid membrane suggests these do not form a tightly associated bundle. The data, however, did indicate weak interactions occur between the FP and TMD, since when they are found in the same membrane, the conformation of both was largely α-helical regardless of the membrane composition [117]. In contrast, using SE-AUC, the FP was found to have a strong interaction with TMD peptides in detergent micelles [118]. Analysis of the FP or TMD alone found that regions of both adopted β-strand conformations in a lipid-dependent manner [105,119]. Furthermore, when the FP and TMD were in the same membrane, a synergistic effect induced significant negative curvature in the membrane. When either the FP or TMD was alone, induction of negative curvature occurred only in membranes that tend towards negative curvature domains (PE membranes). These conflicting results suggest the need for additional analysis of the FP–TMD relationship in paramyxovirus F proteins.

Recent studies have also implicated residues within the TMD as crucial for F protein trafficking and therefore efficient viral particle assembly [120,121,122]. Hendra and Nipah F proteins have a unique trafficking pattern. After synthesis in the ER, the F proteins traffic through the secretory pathway to the plasma membrane as an uncleaved trimer. The F protein is then endocytosed and cleaved in recycling endosomes by the protease cathepsin L before returning back to the surface in its activated pre-fusion form [123,124,125]. Two polar residues within the TMD, S490 and Y498, were shown to be critical for endocytosis and recycling of the F protein, specifically the hydroxyl group of S490 and aromatic ring of Y498. Mutating Y498 decreased trimer association, as judged by association constants from SE-AUC, indicating this residue participates in TMD-TMD associations [121]. Thus, changes in the TMD association may contribute to alterations in intracellular trafficking decisions. Further analysis showed that the proper endocytosis and recycling of the F protein, mediated by residues S490 and Y498, were critical for proper virus-like particle (VLP) formation [120]. This suggests that residues in the TMD participate in viral assembly by facilitating specific intracellular trafficking in Hendra and Nipah viruses, but the extent and mechanism remain unclear, as there are conflicting results on the nature of F trafficking and incorporation in Nipah VLPs [126,127]. Residues in the F protein ectodomain likely also assist in proper F protein incorporation into viral particles, since a chimera of the Rabies virus particle ectodomain and NDV TMD and CTD demonstrated inefficient incorporation into NDV viral particles [122].

Over the past decade, the understanding of the roles of the TMD of paramyxovirus fusion proteins has significantly expanded, demonstrating the critical nature of this region. These studies have shown that the TMDs of paramyxovirus fusion proteins have an active role in both spatial and temporal regulation of the F protein, mediating viral entry, and may be important for efficient viral particle assembly.

### 2.4. Other Class I Viral Fusion Proteins

The above sections review recent findings on the three most intensively studied families of Class I fusion proteins, but additional important studies on the fusion proteins from the *Filoviridae* and *Coronaviridae* families, as well as on Env proteins from retroviruses other than HIV have illuminated the roles of TMDs. The fusion proteins of EBOV and coronaviruses (CoV), such as SARS-CoV and MERS-CoV, are known as GP (for glycoprotein) and S (for Spike), respectively. Both proteins have dual functions in receptor binding and membrane fusion, similar to the HIV Env protein.

The TMDs of both EBOV GP and the SARS-CoV S proteins have been shown to exhibit monomer–trimer–hexamer oligomerization equilibrium when analyzed in isolation by SE-AUC [128]. Analysis of the EBOV GP MPER and TMDs using NMR revealed that both regions appear to be continuous helices independent of the pH, with a turn in between the two adjacent regions [129], similar to the MPER-TMD HIV structure discussed previously [75,76,77]. The MPER of the GP protein appears to lie on the membrane face, with tryptophan and threonine residues mediating contact with the membrane interface. This orientation may represent only one of several potential conformations of the MPER in relation to the TM, as an in situ structure of the full-length EBOV GP protein within the membrane demonstrated that the MPER helices and TMD helices are in line with each other in some conformations of the protein [130]. Biochemical analysis of the GP protein from Marburg virus (MARV), a virus closely related to EBOV, showed MARV GP existed as a monomer in lipid-mimicking environments, further indicating that protein conformations and oligomerization may be dependent on host environmental factors such as lipid composition or pH [131]. While structural analysis has not been completed on the TMDs of CoV S proteins, modeling predictions place a tryptophan-rich region immediately proximal to the TMD [132]. This tryptophan-rich region and its location with respect to the highly hydrophobic coil of the S protein TMD is critical for S-mediated membrane fusion. This study also suggests there is flexibility in the region adjacent to the membrane region [133], with flexibility in the region between the MPER and the TMD critical for large-scale conformational changes of the overall protein. Flexibility between the TMD and the upstream region is characteristic of many Class I fusion proteins, as it has been demonstrated for several different families (Flexible linker IAV, MPER-TMD HIV), though data from paramyxovirus F proteins do not support flexibility for that system [134].

The TMD region of the EBOV GP protein also plays a role in counteracting the host protein tetherin, which can inhibit viral particle release from infected cells [135], in contrast to the mechanism used by HIV-1, which counter-acts tetherin using an accessory viral protein (Vpu) [136]. The first characterization of the role of the EBOV GP protein TMD in counteracting tetherin showed that substituting the TMD of EBOV GP with the Lassa virus GP TMD prohibited EBOV VLPs from inhibiting tetherin activity [137,138]. Further analysis discovered that a GXXXA motif within the EBOV GP protein TMD was responsible for counteracting tetherin activity [139]. This motif was also found to be critical for EBOV filamentous particle release from cells. Mutating this GXXXA motif in EBOV GP decreased viral particle release in a cholesterol-dependent manner [140]. Together, these data suggest a model in which GP trimers, aided by GXXXA motifs within the TMD region, form a lattice along the surface of infected cells in cholesterol rich regions of the membrane. This lattice serves as a particle budding site, eventually closing around actin filaments that are driving this region outward. Particle release then relies in part on this same GXXXA motif interacting with tetherin present within the membrane to counter-act its particle tethering ability.

Using cryo-electron tomography and cryo-electron microscopy (cryo-EM), the structure of full-length Foamy Virus Env was determined [141]. Foamy Virus Env is composed of the gp18 leader peptide, the gp80 surface subunit, which contains the receptor binding site, and the gp48 transmembrane subunit. TMD helices were observed in both the gp48 subunit and the gp18 leader peptide. This structure showed three central coiled helices that were in contact with one another, likely one from each gp48 of the protein trimer. Outside of this coiled-coil, TMD helices from gp18 each appeared to interact with a single helix from the gp48 subunit. Since the TMDs of gp48 likely need to dissociate to mediate membrane fusion, this structure suggests a model in which the gp18 TMD helices block the dissociation of the gp48 until the fusion cascade promotes movement of the entire TMD complex. Computational analysis of the TMD subunits of several Foamy virus strains revealed a conserved lysine–proline motif that suggests a break may exist in the gp48 TMD helix [142], but this was not observed in the structural analysis. However, this motif could serve as a flex point during the conformational changes of the fusion process. Predictions also place a tryptophan-rich region of the protein in the MPER, similar to the MPER in other Class I fusion proteins [75,132].

The fusion proteins (GPs) from arenaviruses have an additional membrane spanning component to consider, the stable signal peptide (SSP). Unlike HIV Env and IAV HA, GPs of arenaviruses form three distinct subunits, G1, responsible for receptor binding, G2, responsible for membrane fusion, and a 58-amino acid SSP [143]. These SSPs have been shown to play a role in the pH-dependent membrane fusion process [144]. Analysis of SSPs has demonstrated they have two membrane-spanning domains, and residues within the membrane participate in interactions with the TMD of the G2 subunit [145,146,147]. These interactions likely serve to prime the G2 subunit for the membrane fusion event.

The past ten years have yielded numerous insights into the TMD of Class I fusion proteins: the first TMD structures have been solved, the active role of this region in several steps of the fusion cascade has been better characterized, and this region has been implicated in protein trafficking and immune function. While these contributions have been groundbreaking, contradictory studies indicate that important work remains to understand this important TMD.

## 3. Class II Fusion Proteins

In 2001, a new class of viral fusion proteins was created because of the large difference in structure of these fusion proteins from Class I fusion proteins [148]. Class II viral fusion proteins include members from the togavirus, flavivirus, and bunyavirus families. While a large amount of α-helical secondary structure is found in Class I fusion proteins, Class II fusion proteins contain three distinct ectodomain regions consisting almost entirely of a β-sheet secondary structure with helical TMDs. These viral fusion proteins associate as homodimers upon synthesis but also form heterodimers with a companion protein. This creates a four-protein complex consisting of two fusion proteins and two companion proteins. Interestingly, for Class II viral fusion proteins, it is the companion protein, not the fusion protein, that requires a proteolytic cleavage event. The companion protein cleavage event primes the fusion protein for the low pH-triggered fusion reaction that occurs in all Class II fusion proteins. To facilitate fusion after exposure to the low pH environment of the endosome, the fusion protein homodimers dissociate into monomers, exposing the previously buried fusion loop, allowing for insertion of this hydrophobic loop into a target membrane. After insertion of the loop into the target membrane, there is a re-association of the monomers into trimers. They remain in a trimeric association throughout the rest of the fusion reaction, completing a series of steps similar to the refolding of the trimer hairpin structure described previously for Class I [149]. In contrast to the extensive work that has been completed on the TMDs of Class I viral fusion proteins over the last decade, only a small number of studies have been performed on the TMDs of Class II viral fusion proteins. These data do, however, provide important structural insight into the TMDs of several Class II fusion proteins [53,150,151,152] and demonstrate a functional role for this region in membrane fusion [153,154].

Using cryo-EM, studies have solved the structures of complete viral particles from Dengue virus, Zika virus (both *Flaviviruses*), and Venezuelan equine encephalitis virus (VEEV) (*Alphavirus*), which include the in situ full-length fusion proteins, E and E1, respectively [53,151,152]. The structure of E proteins for Dengue (Figure 2B) and Zika show two anti-parallel TMD α-helices, as well as three peri-membrane α-helices that lie perpendicular to the TMD helices on the exterior surface of the viral membrane, with each helix connected to the next by a loop [53,151]. Analysis of the Dengue E protein structure showed that the loops of the TMD helices are buried within the head-group region of the inner leaflet of the viral membrane. The two TMD helices form a coiled-coil, with hydrophobic residues facing outward, and multiple serine/threonine hydrophilic residues facing inward on the coiled-coil [53]. These highly hydrophilic interactions appear to have a role similar to the leucine zipper oligomerization motifs found in some Class I fusion proteins [109]. The VEEV E1 protein TMD is composed of two α-helices, with a highly conserved glycine–glycine (GG) kink between them [152]. Since these studies encompassed the full viral particle, they also provide data for TMDs of their respective companion proteins, M for Dengue and Zika and E2 for VEEV. The M protein, similar to E, has two TMD helices that lie anti-parallel to one-another. Despite being situated in close to proximity to the TMD helices of E, the data did not demonstrate any protein-protein contacts between M and E within the membrane space. It was revealed, however, that the presence of both the M and E TMDs provided some lateral order to the membrane, and the membrane was bent to accommodate the short length of the TMD helices, indicating that protein–lipid interactions were likely occurring. The TMD of VEEV E2 was visualized as a long, straight α-helix. When VEEV E1 and E2 were analyzed together, the GG kink in the TMD of E1 appeared to allow TMD flexibility so that the lower portion of the E1 TMD could associate with the E2 TMD helix.

Computational modeling has also provided structural insights into the TMDs of the surface glycoproteins from a pestivirus known as bovine viral diarrhea virus (BVDV) [150]. The two surface glycoproteins of BVDV, E1 and E2, are required for viral entry, with charged residues in the TMD critical for this process [154]. E2 has two TMD α-helices (tmH1 and tmH2), with extensive hydrophobic interactions between them and an arginine residue (R1047) in the loop between the helices which interacts with the phospholipid head groups on the inner leaflet of the membrane. Additionally, hydrogen bonding between the helices at serines S1035 and S1060 may further stabilize the TMD-TMD interactions occurring between tmH1 and tmH2. The TMD of the second surface protein, E1, has two peri-membrane α-helices (pmH1 and pmH2), as well as a single transmembrane α- helix (tmH). As shown by modeling of the E1-E2 hetero-tetramer complex based on ectodomain structural constraints and previously published biochemical constraints, the TMD helix of E1 is in close proximity to the tmH1 of E2, with hydrophobic contacts and a single hydrogen bond (T688 from E1 and Y1056 E2) occurring between the two. This suggests that TMD-TMD associations between E1 and E2 may be important for overall complex formation. Similar to membrane disruptions caused by the TMDs of the Dengue E and M proteins [53], residue R1047 of E2 and charged residues in E1 appear to interact with the phospholipid head groups of the membrane, potentially causing thinning of the membrane which could further assist the viral fusion process. These data together present a model for Class II fusion proteins in which TMD-TMD interactions occur between the fusion protein TMD and the companion protein TMD, with residues in both of these regions interacting with membrane phospholipid headgroups. These interactions may play a role in fusion protein stability and the membrane distortion needed for proper viral entry.

The role of TMD-TMD interactions in the Class II fusion process is also suggested by biochemical studies of the flavivirus E protein, which showed the functional relevance of the second TMD helix [153]. To probe the role of this region in tick-borne encephalitis virus (TBEV), mutants were created with the second TMD helix deleted, or one or both TMD helices substituted with the corresponding helix of the closely related flavivirus, Japanese encephalitis virus (JEV). None of the mutants, even a full deletion of the TMD2, affected early steps in fusion. However, each of these mutants was found to destabilize the post-fusion E trimer which did not allow for the formation of a fusion pore. Interestingly, the chimeras which substituted the full length of both TMDs with a related virus were still able to facilitate the full fusion process, albeit less efficiently than the wild type protein. This suggests a concerted role for interactions between the TMDs in late steps of the fusion pathway, potentially through intra-helix interactions or interactions with the fusion loop in the post-fusion conformation of the protein [153].

While less information is available on Class II fusion proteins compared to recent studies on Class I fusion proteins, critical structural insights into the unique TMDs of these fusion proteins have recently been made. Furthermore, this work has begun to illuminate the extensive TMD-TMD interactions that occur in Class II fusion proteins, potentially similar to those in Class I fusion proteins that may be required for fusion complex formation and complex stability in the fusion process. These recent studies also demonstrate the joint effort of the fusion protein TMD and the TMD of the companion protein in mediating viral entry.

## 4. Class III Fusion Proteins

In 2006, ectodomain structures of fusion proteins from both vesicular stomatitis virus (VSV) [18] and herpes simplex virus type 1 (HSV-1) [13] were solved. The VSV G and HSV-1 gB proteins have remarkably similar structures despite a lack of sequence homology. These fusion protein structures are distinct from both Class I and Class II, prompting the creation of a new class of fusion proteins, Class III [155]. This class now encompasses fusion proteins of viruses in the rhabdovirus, herpesvirus, and baculovirus families [156].

Since 2006, ectodomain structures of the post-fusion forms of HSV-1 gB [13], Epstein–Barr Virus gB [157], and baculovirus gp64 [158] have been solved, and both pre- [19] and post-fusion structures [18] have been solved for VSV G. In the post-fusion form, these fusion proteins are trimeric in nature, similar to both Class I and II proteins. Each fusion protein ectodomain consists of five distinct domains. Starting with domain I that contains fusion loops that lie on the end of extended β-sheet structures, to domain V which forms an α-helical structure that serves as an interacting interface between the proteins in the trimer, and serves to connect the ectodomain with the TMD and cytoplasmic tail portion of these fusion proteins [156]. Unlike the fusion processes in Class I and II, the conformational changes experienced by Class III fusion proteins appear to be reversible in nature. Initial work completed on Class III fusion proteins provided limited insight into the TMD. However, several studies over the past decade have provided structural details and evidence for a role of the TMD in membrane fusion and viral particle assembly.

Recent work demonstrated the crystal structure of a full-length fusion protein from Class III, HSV gB, in its post-fusion form. This structure includes the MPER, TMD, and cytoplasmic tail domain (CTD), all of which were absent in previous structures [54]. The MPER-TMD-CTD regions form a pedestal-like structure through the membrane, with the ectodomain resting on top. The MPER regions of a gB trimer form dynamic helices that lie along the surface of the membrane. At the C-terminus of these helices is a linker with conserved proline and glycine residues that likely contribute to flexibility in this region. C-terminal to this linker lies the TMD, which forms a straight helix that extends through the membrane, with each TMD of the trimer contributing to one side of an inverted teepee structure. The N-terminus of these regions is splayed apart, and each TMD crosses the others at a ~46° angle. The C-terminal portions of the three TMDs do not have cross-promoter linkages, but are in close proximity, in a structure stabilized by knob-and-hole packing and the presence of conserved, small glycine and alanine residues which allow for close fit of the helices. Residues at the C-termini of TMD likely participate in hydrophilic interactions with the CTD. The CTD of each gB monomer in the trimer forms two α-helices (h1a and h2) and one 3_10_ helix (h1b). Immediately following the TMD, h1a and h1b form a zig-zag with proline residues at the interfaces between each helix. Then, an unresolved linker connects to the h2 helix that extends back up toward the membrane. Each CTD region has several contacts with other CTDs of the gB trimer, further stabilizing this base (Figure 2C).

Characterization of the location of mutations within the TMD or CTD previously shown to either enhance or reduce cell–cell fusion allowed for additional insight into gB fusion regulation. Most hyperfusogenic mutants were found to be at residues or regions that would disrupt the trimeric interfaces or the membrane binding of the CTD. Mutants that reduced fusion mainly shorten hydrophobic side chains that participate in interactions at the CTD trimer axis, thus reducing CTD trimer stability and likely causing protein misfolding. Taken together, this suggests that this structure of the CTD also exists in the prefusion conformation of the gB protein, forming a triangular base that may serve as a clamp to hold the TMDs in place until appropriately stimulated, leading to conformational changes required to mediate fusion. This would suggest a model for Class III membrane fusion in which a release of the gB CTD clamp is needed for viral entry to occur, seemingly serving a similar latch role to that of the M protein for flavivirus E [53]. The release of this clamp may be facilitated in part by TMD-TMD interactions between the gH protein and the gB, as work that replaced the gH TMD in pseudorabies virus rendered the virus non-fusogenic [159].

Even before the creation of the new class of fusion proteins, several studies demonstrated the functional role of the TMD of VSV G in membrane fusion [160,161], highlighting specifically that glycine residues in the TMD of VSV G were critical for fusion activity [162]. In the past decade, additional studies have further characterized the contribution of the TMD in rhabdovirus fusion protein function. Substituting the TMD and CTD of rabies virus (RV) fusion protein (G) with similar regions from NDV fusion protein to attempt to aid with incorporation in NDV viral particles as a vaccine vector strategy, showed lower incorporation of this chimera protein in NDV viral particles, despite the chimeric protein having overall cellular protein levels similar to the wild type RV G protein [122,163]. Surface expression of the chimeric protein was not analyzed. Additionally, when mice were vaccinated with NDV particles containing the chimeric RV G protein, virus-induced neutralizing antibodies to rabies virus were lower than those induced by vaccination with NDV particles with the WT RV G protein. This may suggest that successful incorporation into particles relies in part on the TMD or CTD of RV G protein, indicating that signals in the TMD or CTD of RV may play a role in overall particle assembly. These regions may also have a role in the host immune response to viral infection, similar to the production of bNAbs specific to the MPER-TMD produced during HIV infection [77,99].

Analysis of the VSV G TMD in isolation demonstrated a role of this region as a catalyst for membrane fusion [163]. In early stages of membrane fusion (the transition to hemi-fusion), addition of the VSV G TMD increased positive curvature stress and membrane disorder by facilitating acyl-chain movement into the space between the two bilayers. In later stages of fusion, the VSV G TMD created further positive curvature stress at the edges of these induced regions, allowing for the transition between hemi-fusion and fusion pore formation. This suggests that the TMD of VSV G proteins could serve in part to help overcome the large energy barrier to a membrane fusion event by creating physical changes to the membrane.

## 5. Targeting the TMD

As work continues to show the importance of TMDs in viral fusion protein function, it is not surprising that there has also been tremendous growth in the understanding of the role of TMD in the function of other proteins. TMD-TMD interactions are important in the creation of amyloid-β, an important factor in Alzheimer’s disease [164]; the dimerization of both neuropilin-1 [165] and tyrosine kinase receptor ErbB2 [166]; the inhibitory function of the IgG Fc receptor, FcγIIB [167]; and induction of the signaling cascade by frizzled receptors [168]. The growing understanding of a role for TMD-TMD interactions has led to the development of a unique area of therapeutic research studying small molecules that disrupt TMD-TMD interactions [169]. Disrupting the dimerization of neuropilin-1 has been shown to suppress glioma tumor growth in vivo [170]. Plexin-A1 TMD-mimicking peptides disrupt complex formation between neuropilin-1 and Plexin-A1, a complex that forms in gliomas with poor prognoses, and the Plexin-A1 TMD peptides slow both tumor growth and metastasis in cell culture models [171]. Overexpression of p75^NTR^ has been associated with advanced stages of melanoma progression, but a small molecule was able to inhibit tumor growth in a mouse model by targeting the TMD of p75^NTR^ to disrupt oligomerization and downstream signaling of this receptor [172]. Additionally, small molecules targeting the TMD of Epstein–Barr virus latent membrane protein 1 prevent trimerization of the protein, blocking oncogenic activation [173].

TMDs of viral fusion proteins have also been investigated as a novel anti-viral therapeutic strategy. Targeting TMD-TMD interactions as a potential anti-viral therapeutic has been successfully demonstrated for both Class I (paramyxovirus F and HIV Env) and Class II fusion proteins in initial studies. HIV-1 virions that were treated with peptides derived from the FP or TMD of HIV Env exhibited a dose-dependent decrease in infectivity. The Env TMD peptides were able to directly associate with WT full-length Env protein, suggesting that the decrease in virus infectivity is due to the peptide disrupting the native TMD-TMD association [82]. Similarly, co-expression of small proteins mimicking the TMD of the Hendra F protein led to de-stabilization of the full-length F protein, consistent with the presence of additional TMDs reducing the trimeric interactions needed for pre-fusion stability [174]. The TMD proteins also disrupted cell–cell fusion when co-expressed with WT full-length Hendra fusion protein. The ability of TMD peptides to interfere with fusion protein function was also demonstrated in viral infection, as TMD peptides homologous to the TMD of the PIV5 F protein decreased PIV5 infection when incubated with the virus prior to cellular infection. This inhibition was sequence specific, as pre-incubation with PIV5 F TMD peptides did not decrease the infection of a related virus (HMPV) [174]. A similar antiviral strategy was demonstrated using peptides derived from the MPER of Flavivirus E protein. Inclusion of hydrophobic residues corresponding to the TMD of the E protein to the C-terminus of MPER-derived peptides increased the viral inhibitory function of these peptides [175]. These inhibitory effects occurred in a sequence-specific manner, similar to the study completed in paramyxoviruses [174].

Apart from specifically targeting the TMD of fusion proteins as a method to inhibit viral entry, inclusion of the TMD appears to be useful for other viral inhibition strategies. The binding strength of neutralizing antibodies to the MPER region of HIV-1 Env increased when the TMD of the protein was included. Furthermore, when a trimeric TMD was included with the MPER peptide of HIV Env, binding was similar to that of binding the native protein [97,98]. These studies suggest that targeting the TMD or including portions of the TMD in anti-viral therapeutic development may be important.

## 6. Conclusions and Questions

The past decade has provided significant insight into the TMDs of viral fusion proteins from all three classes, further building our understanding of the intricate nature of these regions. Structures have been elucidated for TMDs of each class of fusion proteins, and a function for the TMD in viral fusion has been shown for each class, whether it be in early, intermediate or late stages of the fusion cascade. Significant work has also demonstrated the role of the TMD of fusion proteins outside of the membrane fusion process, implicating it in antibody recognition, host immune responses to viral infection, overall viral particle assembly, and protein trafficking. Despite the large amount of work, critical questions remain unanswered concerning the TMD of viral fusion proteins. How does the conformation of the fusion protein TMD change throughout the membrane fusion cascade? What membrane components influence the TMD for each virus? Are there other interactions between viral or host proteins with the TMD of the fusion protein? Do interactions with the fusion protein TMD exist that influence the overall viral lifecycle? Hopefully, future studies over the next decades will provide answers to these questions.

## References

[1] Earp L.J., Delos S.E., Park H.E., White J.M. (2005). The many mechanisms of viral membrane fusion proteins. Curr. Top. Microbiol. Immunol..

[2] Harrison S.C. (2008). Viral membrane fusion. Nat. Struct. Mol. Biol..

[3] Harrison S.C. (2015). Viral membrane fusion. Virology.

[4] Dutch R.E., Jardetzky T.S., Lamb R.A. (2000). Virus membrane fusion proteins: Biological machines that undergo a metamorphosis. Biosci. Rep..

[5] White J.M., Whittaker G.R. (2016). Fusion of Enveloped Viruses in Endosomes. Traffic.

[6] Kuzmin P.I., Zimmerberg J., Chizmadzhev Y.A., Cohen F.S. (2001). A quantitative model for membrane fusion based on low-energy intermediates. Proc. Natl. Acad. Sci. USA.

[7] Parsegian V.A., Fuller N., Rand R.P. (1979). Measured work of deformation and repulsion of lecithin bilayers. Proc. Natl. Acad. Sci. USA.

[8] Rand R.P., Parsegian V.A. (1984). Physical force considerations in model and biological membranes. Can. J. Biochem. Cell Biol..

[9] White J.M., Delos S.E., Brecher M., Schornberg K. (2008). Structures and mechanisms of viral membrane fusion proteins: Multiple variations on a common theme. Crit. Rev. Biochem. Mol. Biol..

[10] Battles M.B., Mas V., Olmedillas E., Cano O., Vazquez M., Rodriguez L., Melero J.A., McLellan J.S. (2017). Structure and immunogenicity of pre-fusion-stabilized human metapneumovirus F glycoprotein. Nat. Commun..

[11] Bizebard T., Gigant B., Rigolet P., Rasmussen B., Diat O., Bosecke P., Wharton S.A., Skehel J.J., Knossow M. (1995). Structure of influenza virus haemagglutinin complexed with a neutralizing antibody. Nature.

[12] Bullough P.A., Hughson F.M., Skehel J.J., Wiley D.C. (1994). Structure of influenza haemagglutinin at the pH of membrane fusion. Nature.

[13] Heldwein E.E., Lou H., Bender F.C., Cohen G.H., Eisenberg R.J., Harrison S.C. (2006). Crystal structure of glycoprotein B from herpes simplex virus 1. Science.

[14] Julien J.P., Cupo A., Sok D., Stanfield R.L., Lyumkis D., Deller M.C., Klasse P.J., Burton D.R., Sanders R.W., Moore J.P. (2013). Crystal structure of a soluble cleaved HIV-1 envelope trimer. Science.

[15] Kirchdoerfer R.N., Cottrell C.A., Wang N., Pallesen J., Yassine H.M., Turner H.L., Corbett K.S., Graham B.S., McLellan J.S., Ward A.B. (2016). Pre-fusion structure of a human coronavirus spike protein. Nature.

[16] McLellan J.S., Chen M., Leung S., Graepel K.W., Du X., Yang Y., Zhou T., Baxa U., Yasuda E., Beaumont T. (2013). Structure of RSV fusion glycoprotein trimer bound to a prefusion-specific neutralizing antibody. Science.

[17] McLellan J.S., Yang Y., Graham B.S., Kwong P.D. (2011). Structure of respiratory syncytial virus fusion glycoprotein in the postfusion conformation reveals preservation of neutralizing epitopes. J. Virol..

[18] Roche S., Bressanelli S., Rey F.A., Gaudin Y. (2006). Crystal structure of the low-pH form of the vesicular stomatitis virus glycoprotein G. Science.

[19] Roche S., Rey F.A., Gaudin Y., Bressanelli S. (2007). Structure of the prefusion form of the vesicular stomatitis virus glycoprotein G. Science.

[20] Stampfer S.D., Lou H., Cohen G.H., Eisenberg R.J., Heldwein E.E. (2010). Structural basis of local, pH-dependent conformational changes in glycoprotein B from herpes simplex virus type 1. J. Virol..

[21] Weissenhorn W., Dessen A., Harrison S.C., Skehel J.J., Wiley D.C. (1997). Atomic structure of the ectodomain from HIV-1 gp41. Nature.

[22] Wong J.J., Paterson R.G., Lamb R.A., Jardetzky T.S. (2016). Structure and stabilization of the Hendra virus F glycoprotein in its prefusion form. Proc. Natl. Acad. Sci. USA.

[23] Xu K., Chan Y.P., Bradel-Tretheway B., Akyol-Ataman Z., Zhu Y., Dutta S., Yan L., Feng Y., Wang L.F., Skiniotis G. (2015). Crystal Structure of the Pre-fusion Nipah Virus Fusion Glycoprotein Reveals a Novel Hexamer-of-Trimers Assembly. PLoS Pathog..

[24] Yin H.S., Wen X., Paterson R.G., Lamb R.A., Jardetzky T.S. (2006). Structure of the parainfluenza virus 5 F protein in its metastable, prefusion conformation. Nature.

[25] Munro J.B., Mothes W. (2015). Structure and Dynamics of the Native HIV-1 Env Trimer. J. Virol..

[26] Das D.K., Govindan R., Nikic-Spiegel I., Krammer F., Lemke E.A., Munro J.B. (2018). Direct Visualization of the Conformational Dynamics of Single Influenza Hemagglutinin Trimers. Cell.

[27] Benhaim M., Lee K.K. (2018). Single-Molecule Analysis of a Viral Fusion Protein Illuminates a Fusion-Active Intermediate State. Cell.

[28] Lai A.L., Freed J.H. (2015). The Interaction between Influenza HA Fusion Peptide and Transmembrane Domain Affects Membrane Structure. Biophys. J..

[29] Dobrowsky T.M., Zhou Y., Sun S.X., Siliciano R.F., Wirtz D. (2008). Monitoring early fusion dynamics of human immunodeficiency virus type 1 at single-molecule resolution. J. Virol..

[30] Langosch D., Hofmann M., Ungermann C. (2007). The role of transmembrane domains in membrane fusion. Cell Mol. Life. Sci..

[31] Kemble G.W., Danieli T., White J.M. (1994). Lipid-anchored influenza hemagglutinin promotes hemifusion, not complete fusion. Cell.

[32] Melikyan G.B., White J.M., Cohen F.S. (1995). GPI-anchored influenza hemagglutinin induces hemifusion to both red blood cell and planar bilayer membranes. J. Cell Biol..

[33] Tong S., Compans R.W. (1999). Alternative mechanisms of interaction between homotypic and heterotypic parainfluenza virus HN and F proteins. J. Gen. Virol..

[34] Nussler F., Clague M.J., Herrmann A. (1997). Meta-stability of the hemifusion intermediate induced by glycosylphosphatidylinositol-anchored influenza hemagglutinin. Biophys. J..

[35] Armstrong R.T., Kushnir A.S., White J.M. (2000). The transmembrane domain of influenza hemagglutinin exhibits a stringent length requirement to support the hemifusion to fusion transition. J. Cell Biol..

[36] Dong J., Roth M.G., Hunter E. (1992). A chimeric avian retrovirus containing the influenza virus hemagglutinin gene has an expanded host range. J. Virol..

[37] Melikyan G.B., Lin S., Roth M.G., Cohen F.S. (1999). Amino acid sequence requirements of the transmembrane and cytoplasmic domains of influenza virus hemagglutinin for viable membrane fusion. Mol. Biol. Cell.

[38] Roth M.G., Doyle C., Sambrook J., Gething M.J. (1986). Heterologous transmembrane and cytoplasmic domains direct functional chimeric influenza virus hemagglutinins into the endocytic pathway. J. Cell. Biol..

[39] Schroth-Diez B., Ponimaskin E., Reverey H., Schmidt M.F., Herrmann A. (1998). Fusion activity of transmembrane and cytoplasmic domain chimeras of the influenza virus glycoprotein hemagglutinin. J. Virol..

[40] Taylor G.M., Sanders D.A. (1999). The role of the membrane-spanning domain sequence in glycoprotein-mediated membrane fusion. Mol. Biol. Cell.

[41] Langosch D., Heringa J. (1998). Interaction of transmembrane helices by a knobs-into-holes packing characteristic of soluble coiled coils. Proteins.

[42] Markosyan R.M., Cohen F.S., Melikyan G.B. (2000). The lipid-anchored ectodomai of influenza virus hemagglutinin (GPI-HA) is capable of inducing nonenlarging fusion pores. Mol. Biol. Cell.

[43] Chen J., Lee K.H., Steinhauer D.A., Stevens D.J., Skehel J.J., Wiley D.C. (1998). Structure of the hemagglutinin precursor cleavage site, a determinant of influenza pathogenicity and the origin of the labile conformation. Cell.

[44] Chen J., Skehel J.J., Wiley D.C. (1999). N- and C-terminal residues combine in the fusion-pH influenza hemagglutinin HA(2) subunit to form an N cap that terminates the triple-stranded coiled coil. Proc. Natl. Acad. Sci. USA.

[45] Eisen M.B., Sabesan S., Skehel J.J., Wiley D.C. (1997). Binding of the influenza A virus to cell-surface receptors: Structures of five hemagglutinin-sialyloligosaccharide complexes determined by X-ray crystallography. Virology.

[46] Gamblin S.J., Haire L.F., Russell R.J., Stevens D.J., Xiao B., Ha Y., Vasisht N., Steinhauer D.A., Daniels R.S., Elliot A. (2004). The structure and receptor binding properties of the 1918 influenza hemagglutinin. Science.

[47] Sauter N.K., Glick G.D., Crowther R.L., Park S.J., Eisen M.B., Skehel J.J., Knowles J.R., Wiley D.C. (1992). Crystallographic detection of a second ligand binding site in influenza virus hemagglutinin. Proc. Natl. Acad. Sci. USA.

[48] Stevens J., Corper A.L., Basler C.F., Taubenberger J.K., Palese P., Wilson I.A. (2004). Structure of the uncleaved human H1 hemagglutinin from the extinct 1918 influenza virus. Science.

[49] Weis W., Brown J.H., Cusack S., Paulson J.C., Skehel J.J., Wiley D.C. (1988). Structure of the influenza virus haemagglutinin complexed with its receptor, sialic acid. Nature.

[50] Wilson I.A., Skehel J.J., Wiley D.C. (1981). Structure of the haemagglutinin membrane glycoprotein of influenza virus at 3 A resolution. Nature.

[51] Benton D.J., Nans A., Calder L.J., Turner J., Neu U., Lin Y.P., Ketelaars E., Kallewaard N.L., Corti D., Lanzavecchia A. (2018). Influenza hemagglutinin membrane anchor. Proc. Natl. Acad. Sci. USA.

[52] Victor B.L., Baptista A.M., Soares C.M. (2012). Structural determinants for the membrane insertion of the transmembrane peptide of hemagglutinin from influenza virus. J. Chem. Inf. Modeling.

[53] Zhang X., Ge P., Yu X., Brannan J.M., Bi G., Zhang Q., Schein S., Zhou Z.H. (2013). Cryo-EM structure of the mature dengue virus at 3.5-A resolution. Nat. Struct. Mol. Biol..

[54] Cooper R.S., Georgieva E.R., Borbat P.P., Freed J.H., Heldwein E.E. (2018). Structural basis for membrane anchoring and fusion regulation of the herpes simplex virus fusogen gB. Nat. Struct. Mol. Biol..

[55] Benton D.J., Gamblin S.J., Rosenthal P.B., Skehel J.J. (2020). Structural transitions in influenza haemagglutinin at membrane fusion pH. Nature.

[56] Calder L.J., Rosenthal P.B. (2016). Cryomicroscopy provides structural snapshots of influenza virus membrane fusion. Nat. Struct. Mol. Biol..

[57] Chang D.K., Cheng S.F., Kantchev E.A., Lin C.H., Liu Y.T. (2008). Membrane interaction and structure of the transmembrane domain of influenza hemagglutinin and its fusion peptide complex. BMC Biol..

[58] Ge M., Freed J.H. (2011). Two conserved residues are important for inducing highly ordered membrane domains by the transmembrane domain of influenza hemagglutinin. Biophys. J..

[59] Qiao H., Armstrong R.T., Melikyan G.B., Cohen F.S., White J.M. (1999). A specific point mutant at position 1 of the influenza hemagglutinin fusion peptide displays a hemifusion phenotype. Mol. Biol. Cell.

[60] Ranaweera A., Ratnayake P.U., Ekanayaka E.A.P., Declercq R., Weliky D.P. (2019). Hydrogen-Deuterium Exchange Supports Independent Membrane-Interfacial Fusion Peptide and Transmembrane Domains in Subunit 2 of Influenza Virus Hemagglutinin Protein, a Structured and Aqueous-Protected Connection between the Fusion Peptide and Soluble Ectodomain, and the Importance of Membrane Apposition by the Trimer-of-Hairpins Structure. Biochemistry.

[61] Engel S., de Vries M., Herrmann A., Veit M. (2012). Mutation of a raft-targeting signal in the transmembrane region retards transport of influenza virus hemagglutinin through the Golgi. FEBS Lett..

[62] Hu B., Hofer C.T., Thiele C., Veit M. (2019). Cholesterol Binding to the Transmembrane Region of a Group 2 Hemagglutinin (HA) of Influenza Virus Is Essential for Virus Replication, Affecting both Virus Assembly and HA Fusion Activity. J. Virol..

[63] Wilson R.L., Frisz J.F., Klitzing H.A., Zimmerberg J., Weber P.K., Kraft M.L. (2015). Hemagglutinin clusters in the plasma membrane are not enriched with cholesterol and sphingolipids. Biophys. J..

[64] Yang S.T., Kreutzberger A.J.B., Lee J., Kiessling V., Tamm L.K. (2016). The role of cholesterol in membrane fusion. Chem. Phys. Lipids.

[65] Kordyukova L.V., Serebryakova M.V., Baratova L.A., Veit M. (2008). S acylation of the hemagglutinin of influenza viruses: Mass spectrometry reveals site-specific attachment of stearic acid to a transmembrane cysteine. J. Virol..

[66] Scheiffele P., Roth M.G., Simons K. (1997). Interaction of influenza virus haemagglutinin with sphingolipid-cholesterol membrane domains via its transmembrane domain. EMBO J..

[67] Takeda M., Leser G.P., Russell C.J., Lamb R.A. (2003). Influenza virus hemagglutinin concentrates in lipid raft microdomains for efficient viral fusion. Proc. Natl. Acad. Sci. USA.

[68] Veit M., Kretzschmar E., Kuroda K., Garten W., Schmidt M.F., Klenk H.D., Rott R. (1991). Site-specific mutagenesis identifies three cysteine residues in the cytoplasmic tail as acylation sites of influenza virus hemagglutinin. J. Virol..

[69] Wagner R., Herwig A., Azzouz N., Klenk H.D. (2005). Acylation-mediated membrane anchoring of avian influenza virus hemagglutinin is essential for fusion pore formation and virus infectivity. J. Virol..

[70] Barman S., Nayak D.P. (2000). Analysis of the transmembrane domain of influenza virus neuraminidase, a type II transmembrane glycoprotein, for apical sorting and raft association. J. Virol..

[71] Shang L., Yue L., Hunter E. (2008). Role of the membrane-spanning domain of human immunodeficiency virus type 1 envelope glycoprotein in cell-cell fusion and virus infection. J. Virol..

[72] Chen B., Chou J.J. (2017). Structure of the transmembrane domain of HIV-1 envelope glycoprotein. FEBS J..

[73] Dev J., Park D., Fu Q., Chen J., Ha H.J., Ghantous F., Herrmann T., Chang W., Liu Z., Frey G. (2016). Structural basis for membrane anchoring of HIV-1 envelope spike. Science.

[74] Gangupomu V.K., Abrams C.F. (2010). All-atom models of the membrane-spanning domain of HIV-1 gp41 from metadynamics. Biophys. J..

[75] Fu Q., Shaik M.M., Cai Y., Ghantous F., Piai A., Peng H., Rits-Volloch S., Liu Z., Harrison S.C., Seaman M.S. (2018). Structure of the membrane proximal external region of HIV-1 envelope glycoprotein. Proc. Natl. Acad. Sci. USA.

[76] Kwon B., Lee M., Waring A.J., Hong M. (2018). Oligomeric Structure and Three-Dimensional Fold of the HIV gp41 Membrane-Proximal External Region and Transmembrane Domain in Phospholipid Bilayers. J. Am. Chem. Soc..

[77] Apellaniz B., Rujas E., Serrano S., Morante K., Tsumoto K., Caaveiro J.M., Jimenez M.A., Nieva J.L. (2015). The Atomic Structure of the HIV-1 gp41 Transmembrane Domain and Its Connection to the Immunogenic Membrane-proximal External Region. J. Biol. Chem..

[78] Buzon V., Natrajan G., Schibli D., Campelo F., Kozlov M.M., Weissenhorn W. (2010). Crystal Structure of HIV-1 gp41 Including Both Fusion Peptide and Membrane Proximal External Regions. PLoS Pathog..

[79] Lee J.H., Ozorowski G., Ward A.B. (2016). Cryo-EM structure of a native, fully glycosylated, cleaved HIV-1 envelope trimer. Science.

[80] Reardon P.N., Sage H., Dennison S.M., Martin J.W., Donald B.R., Alam S.M., Haynes B.F., Spicer L.D. (2014). Structure of an HIV-1-neutralizing antibody target, the lipid-bound gp41 envelope membrane proximal region trimer. Proc. Natl. Acad. Sci. USA.

[81] Zanetti G., Briggs J.A., Grunewald K., Sattentau Q.J., Fuller S.D. (2006). Cryo-electron tomographic structure of an immunodeficiency virus envelope complex in situ. PLoS Pathog..

[82] Reuven E.M., Dadon Y., Viard M., Manukovsky N., Blumenthal R., Shai Y. (2012). HIV-1 gp41 transmembrane domain interacts with the fusion peptide: Implication in lipid mixing and inhibition of virus-cell fusion. Biochemistry.

[83] Lee M., Morgan C.A., Hong M. (2019). Fully hydrophobic HIV gp41 adopts a hemifusion-like conformation in phospholipid bilayers. J. Biol. Chem..

[84] Kondo N., Miyauchi K., Meng F., Iwamoto A., Matsuda Z. (2010). Conformational changes of the HIV-1 envelope protein during membrane fusion are inhibited by the replacement of its membrane-spanning domain. J. Biol. Chem..

[85] Long Y., Meng F., Kondo N., Iwamoto A., Matsuda Z. (2011). Conserved arginine residue in the membrane-spanning domain of HIV-1 gp41 is required for efficient membrane fusion. Protein Cell.

[86] Hollingsworth L.R.t., Lemkul J.A., Bevan D.R., Brown A.M. (2018). HIV-1 Env gp41 Transmembrane Domain Dynamics Are Modulated by Lipid, Water, and Ion Interactions. Biophys. J..

[87] Baker M.K., Gangupomu V.K., Abrams C.F. (2014). Characterization of the water defect at the HIV-1 gp41 membrane spanning domain in bilayers with and without cholesterol using molecular simulations. Biochim. Biophys. Acta.

[88] Piai A., Dev J., Fu Q., Chou J.J. (2017). Stability and Water Accessibility of the Trimeric Membrane Anchors of the HIV-1 Envelope Spikes. J. Am. Chem. Soc..

[89] Ashkenazi A., Faingold O., Kaushansky N., Ben-Nun A., Shai Y. (2013). A highly conserved sequence associated with the HIV gp41 loop region is an immunomodulator of antigen-specific T cells in mice. Blood.

[90] Bloch I., Quintana F.J., Gerber D., Cohen T., Cohen I.R., Shai Y. (2007). T-cell inactivation and immunosuppressive activity induced by HIV gp41 via novel interacting motif. FASEB J..

[91] Rotem E., Reuven E.M., Klug Y.A., Shai Y. (2016). The Transmembrane Domain of HIV-1 gp41 Inhibits T-Cell Activation by Targeting Multiple T-Cell Receptor Complex Components through Its GxxxG Motif. Biochemistry.

[92] Klug Y.A., Rotem E., Schwarzer R., Shai Y. (2017). Mapping out the intricate relationship of the HIV envelope protein and the membrane environment. Biochim. Biophys. Acta Biomembr..

[93] Reuven E.M., Ali M., Rotem E., Schwarzer R., Gramatica A., Futerman A.H., Shai Y. (2014). The HIV-1 envelope transmembrane domain binds TLR2 through a distinct dimerization motif and inhibits TLR2-mediated responses. PLoS Pathog..

[94] Faingold O., Cohen T., Shai Y. (2012). A GxxxG-like motif within HIV-1 fusion peptide is critical to its immunosuppressant activity, structure, and interaction with the transmembrane domain of the T-cell receptor. J. Biol. Chem..

[95] Cohen T., Pevsner-Fischer M., Cohen N., Cohen I.R., Shai Y. (2008). Characterization of the interacting domain of the HIV-1 fusion peptide with the transmembrane domain of the T-cell receptor. Biochemistry.

[96] Quintana F.J., Gerber D., Kent S.C., Cohen I.R., Shai Y. (2005). HIV-1 fusion peptide targets the TCR and inhibits antigen-specific T cell activation. J. Clin. Invest..

[97] Reichart T.M., Baksh M.M., Rhee J.K., Fiedler J.D., Sligar S.G., Finn M.G., Zwick M.B., Dawson P.E. (2016). Trimerization of the HIV Transmembrane Domain in Lipid Bilayers Modulates Broadly Neutralizing Antibody Binding. Angew. Chem. Int. Ed. Engl..

[98] Torrents de la Pena A., Rantalainen K., Cottrell C.A., Allen J.D., van Gils M.J., Torres J.L., Crispin M., Sanders R.W., Ward A.B. (2019). Similarities and differences between native HIV-1 envelope glycoprotein trimers and stabilized soluble trimer mimetics. PLoS Pathog..

[99] Wang Y., Kaur P., Sun Z.J., Elbahnasawy M.A., Hayati Z., Qiao Z.S., Bui N.N., Chile C., Nasr M.L., Wagner G. (2019). Topological analysis of the gp41 MPER on lipid bilayers relevant to the metastable HIV-1 envelope prefusion state. Proc. Natl. Acad. Sci. USA.

[100] Pinto D., Fenwick C., Caillat C., Silacci C., Guseva S., Dehez F., Chipot C., Barbieri S., Minola A., Jarrossay D. (2019). Structural Basis for Broad HIV-1 Neutralization by the MPER-Specific Human Broadly Neutralizing Antibody LN01. Cell Host Microbe.

[101] Checkley M.A., Luttge B.G., Freed E.O. (2011). HIV-1 envelope glycoprotein biosynthesis, trafficking, and incorporation. J. Mol. Biol..

[102] Miyauchi K., Curran A.R., Long Y., Kondo N., Iwamoto A., Engelman D.M., Matsuda Z. (2010). The membrane-spanning domain of gp41 plays a critical role in intracellular trafficking of the HIV envelope protein. Retrovirology.

[103] Perrin J., Bary A., Vernay A., Cosson P. (2018). Role of the HIV-1 envelope transmembrane domain in intracellular sorting. BMC Cell Biol..

[104] Lee M., Yao H., Kwon B., Waring A.J., Ruchala P., Singh C., Hong M. (2018). Conformation and Trimer Association of the Transmembrane Domain of the Parainfluenza Virus Fusion Protein in Lipid Bilayers from Solid-State NMR: Insights into the Sequence Determinants of Trimer Structure and Fusion Activity. J. Mol. Biol..

[105] Yao H., Lee M.W., Waring A.J., Wong G.C., Hong M. (2015). Viral fusion protein transmembrane domain adopts beta-strand structure to facilitate membrane topological changes for virus-cell fusion. Proc. Natl. Acad. Sci. USA.

[106] Bissonnette M.L., Donald J.E., DeGrado W.F., Jardetzky T.S., Lamb R.A. (2009). Functional analysis of the transmembrane domain in paramyxovirus F protein-mediated membrane fusion. J. Mol. Biol..

[107] Afonso C.L., Amarasinghe G.K., Banyai K., Bao Y., Basler C.F., Bavari S., Bejerman N., Blasdell K.R., Briand F.X., Briese T. (2016). Taxonomy of the order Mononegavirales: Update 2016. Arch. Virol..

[108] Smith E.C., Smith S.E., Carter J.R., Webb S.R., Gibson K.M., Hellman L.M., Fried M.G., Dutch R.E. (2013). Trimeric transmembrane domain interactions in paramyxovirus fusion proteins: Roles in protein folding, stability, and function. J. Biol. Chem..

[109] Webb S., Nagy T., Moseley H., Fried M., Dutch R. (2017). Hendra virus fusion protein transmembrane domain contributes to pre-fusion protein stability. J. Biol. Chem..

[110] Gravel K.A., McGinnes L.W., Reitter J., Morrison T.G. (2011). The transmembrane domain sequence affects the structure and function of the Newcastle disease virus fusion protein. J. Virol..

[111] Branttie J.M., Dutch R.E. (2020). Parainfluenza virus 5 fusion protein maintains pre-fusion stability but not fusogenic activity following mutation of a transmembrane leucine/isoleucine domain. J. Gen. Virol..

[112] Slaughter K.B., Dutch R.E. (2019). Transmembrane Domain Dissociation Is Required for Hendra Virus F Protein Fusogenic Activity. J. Virol..

[113] Smith E.C., Culler M.R., Hellman L.M., Fried M.G., Creamer T.P., Dutch R.E. (2012). Beyond anchoring: The expanding role of the hendra virus fusion protein transmembrane domain in protein folding, stability, and function. J. Virol..

[114] Zokarkar A., Connolly S.A., Jardetzky T.S., Lamb R.A. (2012). Reversible Inhibition of Fusion Activity of a Paramyxovirus Fusion Protein by an Engineered Disulfide Bond in the Membrane-Proximal External Region. J. Virol..

[115] Eckert D.M., Kim P.S. (2001). Mechanisms of viral membrane fusion and its inhibition. Annu. Rev. Biochem..

[116] Plattet P., Plemper R.K. (2013). Envelope protein dynamics in paramyxovirus entry. mBio.

[117] Yao H., Lee M., Liao S.Y., Hong M. (2016). Solid-State Nuclear Magnetic Resonance Investigation of the Structural Topology and Lipid Interactions of a Viral Fusion Protein Chimera Containing the Fusion Peptide and Transmembrane Domain. Biochemistry.

[118] Donald J.E., Zhang Y., Fiorin G., Carnevale V., Slochower D.R., Gai F., Klein M.L., DeGrado W.F. (2011). Transmembrane orientation and possible role of the fusogenic peptide from parainfluenza virus 5 (PIV5) in promoting fusion. Proc. Natl. Acad. Sci. USA.

[119] Weise K., Reed J. (2008). Fusion peptides and transmembrane domains of fusion proteins are characterized by different but specific structural properties. Chembiochem.

[120] Cifuentes-Munoz N., Sun W., Ray G., Schmitt P.T., Webb S., Gibson K., Dutch R.E., Schmitt A.P. (2017). Mutations in the Transmembrane Domain and Cytoplasmic Tail of Hendra Virus Fusion Protein Disrupt Virus-Like-Particle Assembly. J. Virol..

[121] Popa A., Carter J.R., Smith S.E., Hellman L., Fried M.G., Dutch R.E. (2012). Residues in the hendra virus fusion protein transmembrane domain are critical for endocytic recycling. J. Virol..

[122] Yu G.M., Zu S.L., Zhou W.W., Wang X.J., Shuai L., Wang X.L., Ge J.Y., Bu Z.G. (2017). Chimeric rabies glycoprotein with a transmembrane domain and cytoplasmic tail from Newcastle disease virus fusion protein incorporates into the Newcastle disease virion at reduced levels. J. Vet. Sci..

[123] Pager C.T., Craft W.W., Patch J., Dutch R.E. (2006). A mature and fusogenic form of the Nipah virus fusion protein requires proteolytic processing by cathepsin L.. Virology.

[124] Pager C.T., Dutch R.E. (2005). Cathepsin L is involved in proteolytic processing of the Hendra virus fusion protein. J. Virol..

[125] Pager C.T., Wurth M.A., Dutch R.E. (2004). Subcellular localization and calcium and pH requirements for proteolytic processing of the Hendra virus fusion protein. J. Virol..

[126] Liu Q., Chen L., Aguilar H.C., Chou K.C. (2018). A stochastic assembly model for Nipah virus revealed by super-resolution microscopy. Nat. Commun..

[127] Mattera R., Farias G.G., Mardones G.A., Bonifacino J.S. (2014). Co-assembly of viral envelope glycoproteins regulates their polarized sorting in neurons. PLoS Pathog..

[128] Webb S.R., Smith S.E., Fried M.G., Dutch R.E. (2018). Transmembrane Domains of Highly Pathogenic Viral Fusion Proteins Exhibit Trimeric Association In Vitro. mSphere.

[129] Lee J., Nyenhuis D.A., Nelson E.A., Cafiso D.S., White J.M., Tamm L.K. (2017). Structure of the Ebola virus envelope protein MPER/TM domain and its interaction with the fusion loop explains their fusion activity. Proc. Natl. Acad. Sci. USA.

[130] Beniac D.R., Booth T.F. (2017). Structure of the Ebola virus glycoprotein spike within the virion envelope at 11 A resolution. Sci. Rep..

[131] Liu N., Girvin M.E., Brenowitz M., Lai J.R. (2019). Conformational and lipid bilayer-perturbing properties of Marburg virus GP2 segments containing the fusion loop and membrane-proximal external region/transmembrane domain. Heliyon.

[132] Corver J., Broer R., van Kasteren P., Spaan W. (2009). Mutagenesis of the transmembrane domain of the SARS coronavirus spike glycoprotein: Refinement of the requirements for SARS coronavirus cell entry. Virol. J..

[133] Kawase M., Kataoka M., Shirato K., Matsuyama S. (2019). Biochemical Analysis of Coronavirus Spike Glycoprotein Conformational Intermediates during Membrane Fusion. J. Virol..

[134] Baker K.A., Dutch R.E., Lamb R.A., Jardetzky T.S. (1999). Structural basis for paramyxovirus-mediated membrane fusion. Mol. Cell.

[135] Neil S.J. (2013). The antiviral activities of tetherin. Curr. Top. Microbiol. Immunol..

[136] Neil S.J., Zang T., Bieniasz P.D. (2008). Tetherin inhibits retrovirus release and is antagonized by HIV-1 Vpu. Nature.

[137] Gnirss K., Fiedler M., Kramer-Kuhl A., Bolduan S., Mittler E., Becker S., Schindler M., Pohlmann S. (2014). Analysis of determinants in filovirus glycoproteins required for tetherin antagonism. Viruses.

[138] Vande Burgt N.H., Kaletsky R.L., Bates P. (2015). Requirements within the Ebola Viral Glycoprotein for Tetherin Antagonism. Viruses.

[139] Gonzalez-Hernandez M., Hoffmann M., Brinkmann C., Nehls J., Winkler M., Schindler M., Pohlmann S. (2018). A GXXXA Motif in the Transmembrane Domain of the Ebola Virus Glycoprotein Is Required for Tetherin Antagonism. J. Virol..

[140] Hacke M., Bjorkholm P., Hellwig A., Himmels P., Ruiz de Almodovar C., Brugger B., Wieland F., Ernst A.M. (2015). Inhibition of Ebola virus glycoprotein-mediated cytotoxicity by targeting its transmembrane domain and cholesterol. Nat. Commun..

[141] Effantin G., Estrozi L.F., Aschman N., Renesto P., Stanke N., Lindemann D., Schoehn G., Weissenhorn W. (2016). Cryo-electron Microscopy Structure of the Native Prototype Foamy Virus Glycoprotein and Virus Architecture. PLoS Pathog..

[142] Wang M., Zhang H., Liu Q.M., Sun Y., Li Z., Liu W.H., He X.H., Song J., Wang Y.X. (2016). Structure of transmembrane subunits gp47 of the foamy virus envelope glycoproteins. Acta Virol..

[143] York J., Romanowski V., Lu M., Nunberg J.H. (2004). The signal peptide of the Junín arenavirus envelope glycoprotein is myristoylated and forms an essential subunit of the mature G1-G2 complex. J. Virol..

[144] York J., Nunberg J.H. (2006). Role of the stable signal peptide of Junín arenavirus envelope glycoprotein in pH-dependent membrane fusion. J. Virol..

[145] Agnihothram S.S., York J., Trahey M., Nunberg J.H. (2007). Bitopic membrane topology of the stable signal peptide in the tripartite Junín virus GP-C envelope glycoprotein complex. J. Virol..

[146] Messina E.L., York J., Nunberg J.H. (2012). Dissection of the role of the stable signal peptide of the arenavirus envelope glycoprotein in membrane fusion. J. Virol..

[147] York J., Nunberg J.H. (2009). Intersubunit interactions modulate pH-induced activation of membrane fusion by the Junin virus envelope glycoprotein GPC. J. Virol..

[148] Lescar J., Roussel A., Wien M.W., Navaza J., Fuller S.D., Wengler G., Wengler G., Rey F.A. (2001). The Fusion Glycoprotein Shell of Semliki Forest Virus. Cell.

[149] Kielian M. (2006). Class II virus membrane fusion proteins. Virology.

[150] Wang J., Li Y., Modis Y. (2014). Structural models of the membrane anchors of envelope glycoproteins E1 and E2 from pestiviruses. Virology.

[151] Sirohi D., Chen Z., Sun L., Klose T., Pierson T.C., Rossmann M.G., Kuhn R.J. (2016). The 3.8 A resolution cryo-EM structure of Zika virus. Science.

[152] Zhang R., Hryc C.F., Cong Y., Liu X., Jakana J., Gorchakov R., Baker M.L., Weaver S.C., Chiu W. (2011). 4.4 A cryo-EM structure of an enveloped alphavirus Venezuelan equine encephalitis virus. EMBO J..

[153] Fritz R., Blazevic J., Taucher C., Pangerl K., Heinz F.X., Stiasny K. (2011). The unique transmembrane hairpin of flavivirus fusion protein E is essential for membrane fusion. J. Virol..

[154] Ronecker S., Zimmer G., Herrler G., Greiser-Wilke I., Grummer B. (2008). Formation of bovine viral diarrhea virus E1-E2 heterodimers is essential for virus entry and depends on charged residues in the transmembrane domains. J. Gen. Virol..

[155] Steven A.C., Spear P.G. (2006). Biochemistry. Viral glycoproteins and an evolutionary conundrum. Science.

[156] Backovic M., Jardetzky T.S. (2009). Class III viral membrane fusion proteins. Curr. Opin. Struct. Biol..

[157] Backovic M., Longnecker R., Jardetzky T.S. (2009). Structure of a trimeric variant of the Epstein-Barr virus glycoprotein B. Proc. Natl. Acad. Sci. USA.

[158] Kadlec J., Loureiro S., Abrescia N.G., Stuart D.I., Jones I.M. (2008). The postfusion structure of baculovirus gp64 supports a unified view of viral fusion machines. Nat. Struct. Mol. Biol..

[159] Vallbracht M., Fuchs W., Klupp B.G., Mettenleiter T.C. (2018). Functional Relevance of the Transmembrane Domain and Cytoplasmic Tail of the Pseudorabies Virus Glycoprotein H for Membrane Fusion. J. Virol..

[160] Dennison S.M., Greenfield N., Lenard J., Lentz B.R. (2002). VSV Transmembrane Domain (TMD) Peptide Promotes PEG-Mediated Fusion of Liposomes in a Conformationally Sensitive Fashion†. Biochemistry.

[161] Odell D., Wanas E., Yan J., Ghosh H.P. (1997). Influence of membrane anchoring and cytoplasmic domains on the fusogenic activity of vesicular stomatitis virus glycoprotein G. J. Virol..

[162] Cleverley D.Z., Lenard J. (1998). The transmembrane domain in viral fusion: Essential role for a conserved glycine residue in vesicular stomatitis virus G protein. Proc. Natl. Acad. Sci. USA.

[163] Sengupta T., Chakraborty H., Lentz B.R. (2014). The transmembrane domain peptide of vesicular stomatitis virus promotes both intermediate and pore formation during PEG-mediated vesicle fusion. Biophys. J..

[164] Abad C., Martinez-Gil L., Tamborero S., Mingarro I. (2009). Membrane topology of gp41 and amyloid precursor protein: Interfering transmembrane interactions as potential targets for HIV and Alzheimer treatment. Biochim. Biophys. Acta.

[165] Roth L., Nasarre C., Dirrig-Grosch S., Aunis D., Cremel G., Hubert P., Bagnard D. (2008). Transmembrane domain interactions control biological functions of neuropilin-1. Mol. Biol. Cell.

[166] Arpel A., Sawma P., Spenle C., Fritz J., Meyer L., Garnier N., Velazquez-Quesada I., Hussenet T., Aci-Seche S., Baumlin N. (2014). Transmembrane domain targeting peptide antagonizing ErbB2/Neu inhibits breast tumor growth and metastasis. Cell Rep..

[167] Wang J., Li Z., Xu L., Yang H., Liu W. (2018). Transmembrane domain dependent inhibitory function of FcgammaRIIB. Protein Cell.

[168] Zhang W., Lu W., Ananthan S., Suto M.J., Li Y. (2017). Discovery of novel frizzled-7 inhibitors by targeting the receptor’s transmembrane domain. Oncotarget.

[169] Pessi A., Langella A., Capito E., Ghezzi S., Vicenzi E., Poli G., Ketas T., Mathieu C., Cortese R., Horvat B. (2012). A general strategy to endow natural fusion-protein-derived peptides with potent antiviral activity. PLoS ONE.

[170] Nasarre C., Roth M., Jacob L., Roth L., Koncina E., Thien A., Labourdette G., Poulet P., Hubert P., Cremel G. (2010). Peptide-based interference of the transmembrane domain of neuropilin-1 inhibits glioma growth in vivo. Oncogene.

[171] Jacob L., Sawma P., Garnier N., Meyer L.A., Fritz J., Hussenet T., Spenle C., Goetz J., Vermot J., Fernandez A. (2016). Inhibition of PlexA1-mediated brain tumor growth and tumor-associated angiogenesis using a transmembrane domain targeting peptide. Oncotarget.

[172] Goh E.T.H., Lin Z., Ahn B.Y., Lopes-Rodrigues V., Dang N.H., Salim S., Berger B., Dymock B., Senger D.L., Ibanez C.F. (2018). A Small Molecule Targeting the Transmembrane Domain of Death Receptor p75(NTR) Induces Melanoma Cell Death and Reduces Tumor Growth. Cell Chem. Biol..

[173] Wang X., Saludes J.P., Zhao T.X., Csakai A., Fiorini Z., Chavez S.A., Li J., Lee G.I., Varga K., Yin H. (2012). Targeting the lateral interactions of transmembrane domain 5 of Epstein-Barr virus latent membrane protein 1. Biochim. Biophys. Acta.

[174] Barrett C.T., Webb S.R., Dutch R.E. (2019). A Hydrophobic Target: Using the Paramyxovirus Fusion Protein Transmembrane Domain To Modulate Fusion Protein Stability. J. Virol..

[175] Schmidt A.G., Yang P.L., Harrison S.C. (2010). Peptide inhibitors of flavivirus entry derived from the E protein stem. J. Virol..

